# Supplementary material for: Message framing to inform cancer prevention pricing interventions in the UK and USA: a factorial experiment, 2019
Source: BMJ Open. 2021 Jan 25;11(1):e041324. doi: 10.1136/bmjopen-2020-041324 (PMC7839858; doi:10.1136/bmjopen-2020-041324)
Supplement: Author's manuscript [file bmjopen-2020-041324.draft_revisions.pdf]

BMJ Open

BMJ Open is committed to open peer review. As part of this commitment we make the peer review history of every article we publish publicly available.

When an article is published we post the peer reviewers' comments and the authors' responses online. We also post the versions of the paper that were used during peer review. These are the versions that the peer review comments apply to.

The versions of the paper that follow are the versions that were submitted during the peer review process. They are not the versions of record or the final published versions. They should not be cited or distributed as the published version of this manuscript.

BMJ Open is an open access journal and the full, final, typeset and author-corrected version of record of the manuscript is available on our site with no access controls, subscription charges or pay-per-view fees (http://bmjopen.bmj.com).

If you have any questions on BMJ Open's open peer review process please email info.bmjopen@bmj.com

BMJ Open

Message framing to inform cancer prevention pricing interventions in the UK and US: A factorial experiment, 2019

Journal:	*BMJ Open*
Manuscript ID	bmjopen-2020-041324
Article Type:	Original research
Date Submitted by the Author:	04-Jun-2020

Complete List of Authors:	Lee, Joseph; East Carolina University, Department of Health Education and Promotion, College of Health and Human Performance; University of North Carolina Lineberger Comprehensive Cancer Center, Cancer Prevention and Control<br>Cristello, Julie; Florida International University,<br>Buckton, Christina; University of Glasgow, MRC/CSO Social and Public Health Sciences Unit, Institute of Health & Wellbeing<br>Carey, Rachel; University College London, Department of Clinical, Educational & Health Psychology<br>Trucco, Elisa; Florida International University, Department of Psychology and Center for Children and Families; University of Michigan, Department of Psychiatry<br>Schenk, Paulina; University College London, Department of Clinical, Educational & Health Psychology<br>Ikegwuonu, Theresa; University of Glasgow, MRC/CSO Social and Public Health Sciences Unit, Institute of Health & Wellbeing<br>Hilton , Shona; University of Glasgow, MRC/CSO Social and Public Health Sciences Unit, Institute of Health & Wellbeing<br>Golden, SD; University of North Carolina at Chapel Hill Gillings School of Global Public Health; University of North Carolina Lineberger Comprehensive Cancer Center, Cancer Prevention and Control<br>Conway, D; University of Glasgow, School of Medicine, Dentistry, and Nursing, College of Medical Veterinary and Life Sciences

Keywords:	Health policy < HEALTH SERVICES ADMINISTRATION & MANAGEMENT, PREVENTIVE MEDICINE, PUBLIC HEALTH

SCHOLARONE™
Manuscripts

I, the Submitting Author has the right to grant and does grant on behalf of all authors of the Work (as defined in the below author licence), an exclusive licence and/or a non-exclusive licence for contributions from authors who are: i) UK Crown employees; ii) where BMJ has agreed a CC-BY licence shall apply, and/or iii) in accordance with the terms applicable for US Federal Government officers or employees acting as part of their official duties; on a worldwide, perpetual, irrevocable, royalty-free basis to BMJ Publishing Group Ltd ("BMJ") its licensees and where the relevant Journal is co-owned by BMJ to the co-owners of the Journal, to publish the Work in this journal and any other BMJ products and to exploit all rights, as set out in our licence.

The Submitting Author accepts and understands that any supply made under these terms is made by BMJ to the Submitting Author unless you are acting as an employee on behalf of your employer or a postgraduate student of an affiliated institution which is paying any applicable article publishing charge ("APC") for Open Access articles. Where the Submitting Author wishes to make the Work available on an Open Access basis (and intends to pay the relevant APC), the terms of reuse of such Open Access shall be governed by a Creative Commons licence – details of these licences and which Creative Commons licence will apply to this Work are set out in our licence referred to above.

Other than as permitted in any relevant BMJ Author's Self Archiving Policies, I confirm this Work has not been accepted for publication elsewhere, is not being considered for publication elsewhere and does not duplicate material already published. I confirm all authors consent to publication of this Work and authorise the granting of this licence.

For peer review only

Public Health

For submission to: *BMJ Open*

Message framing to inform cancer prevention pricing interventions in the UK and US: A factorial experiment, 2019

Joseph G. L. Lee, PhD, MPH,[1,2*] Julie V. Cristello, MS,[3,4] Christina H. Buckton, MSc, MBA,[5] Rachel N. Carey, PhD,[6] Elisa M. Trucco, PhD,[3,4,7] Paulina M. Schenk, MSc,[6] Theresa Ikegwuonu, PhD,[5] Prof. Shona Hilton, PhD,[5] Shelley D. Golden, PhD, MPH,[2,8] Prof. David I. Conway, BDS, FDS, MPH, FFPH, PhD[9]

1. Department of Health Education and Promotion, College of Health and Human Performance, East Carolina University, Greenville, NC, USA

2. Cancer Prevention and Control, UNC Lineberger Comprehensive Cancer Center, Chapel Hill, NC, USA

3. Department of Psychology, Florida International University, Miami, FL, USA

4. Center for Children and Families, Florida International University, Miami, FL, USA

5. MRC/CSO Social and Public Health Sciences Unit, Institute of Health & Wellbeing, University of Glasgow, Glasgow, UK

6. Department of Clinical, Educational & Health Psychology, University College London, London, UK

7. Department of Psychiatry, University of Michigan, Ann Arbor, MI, USA

8. Department of Health Behavior, UNC Gillings School of Global Public Health, Chapel Hill, NC, USA

9. School of Medicine, Dentistry, and Nursing, College of Medical Veterinary and Life Sciences, University of Glasgow, Glasgow, UK

* Corresponding author: Dr. Joseph G. L. Lee, Department of Health Education and Promotion, East Carolina University, 1000 East Fifth Street Stop 529, Greenville, NC 27858; phone: +1 (252) 328-4661; fax: +1 (252) 328-1285; leejose14@ecu.edu

Key Words: public policy, policy making, prevention and control, taxes, neoplasms
Word Count: 4,120

For peer review only - http://bmjopen.bmj.com/site/about/guidelines.xhtml

Abstract (293/300 words)

Objectives: To advance understanding of how message framing can be used to maximise public support across different pricing policies for alcohol, tobacco, and sugar products that prevent consumption of cancer-causing products.

Design: We designed a 3x4x3 randomised factorial experiment to test responses to messages with three pricing policies, four message frames, and three products.

Setting: Online survey panel (Qualtrics) in 2019.

Participants: Adults (N=1850) from the UK and USA.

Interventions: Participants randomly viewed one of 36 separate messages that varied by pricing policy (increasing taxes, getting rid of price discounts, getting rid of low-cost products), four frames, and product (alcohol, tobacco, sugar).

Primary and secondary outcome measures: We assessed the relationship between the message characteristics and four dependent variables. Three were related to policy support: (1) increasing taxes on the product mentioned in the message, (2) getting rid of price discounts and special offers on the product mentioned in the message, (3) getting rid of low-cost versions of the product mentioned in the message. One was related to reactance, a psychological response to having one's freedom limited and used six items.

Results: We found no effect for pricing policy in the message. Frames regarding children and reducing cancer risk moderated some outcomes, showing promise for real-world use. We found differences in support by product and reactance with greatest support and least reactance for tobacco policies, less support and more reactance for alcohol policies, and the least support and most reactance for sugar policies.

Conclusions: Cancer prevention efforts utilizing policy interventions can be informed by the message framing literature. Our results offer insights for cancer prevention advocacy efforts across the UK and USA and highlight that tax versus non-tax approaches to increasing the cost of cancer-causing products result in similar responses from consumers.

Article Summary

Strengths and limitations of this study

- We conducted qualitative formative work and a pilot study to inform the experimental design and stimuli.
- Our team and data represented both the UK and the USA.
- We have strong internal validity given our use of a randomised factorial design.
- Our sample size was based on a power calculation for small effect sizes.
- Testing messages in a controlled experiment likely has limited ecological validity given real-world exposure to multiple messages across multiple channels.

For peer review only - http://bmjopen.bmj.com/site/about/guidelines.xhtml

Funding

This work was supported by Cancer Research UK (C18486/A25644), internal research funds at East Carolina University, UK Medical Research Council (MC_UU_12017/13, MC_UU_12017/15), the Chief Scientist Office of the Scottish Government Health Directorates (SPHSU15), and the U.S. National Institutes of Health (K08AA023290, T32DA043449). The content is solely the responsibility of the authors and does not necessarily represent the official views of the funders.

Competing Interests

The authors report no competing interests.

Data Sharing

Data, codebooks, and interview guides are available in East Carolina University Dataverse, doi:10.15139/S3/IAKUQN, available from: https://dataverse.unc.edu/dataset.xhtml?persistentId=doi:10.15139/S3/IAKUQN

For peer review only

For peer review only - http://bmjopen.bmj.com/site/about/guidelines.xhtml

INTRODUCTION

One-third of the risk and burden of cancer is attributed to four common behaviours – tobacco use, use of alcohol, poor dietary choices, and physical inactivity.[1] These individual behaviours are influenced by a complex array of factors, including people's perceptions, beliefs as well as macro-level drivers such as corporate marketing of unhealthy products.[2,3] Pricing interventions that increase the price per unit of harmful products are an effective measure governments have to help encourage consumers to reduce the consumption of products that contribute to cancer.[4-12] There are multiple ways to increase per-unit costs of products. Many tobacco, alcohol and sugar-sweetened products can be subject to excise taxes, which levy a specific or proportional fee on a product that is collected as revenue by the levying government. In addition to taxation, emerging research identifies *non-tax* price-raising strategies, including policies that set a minimum product price, mitigation fees that recoup public costs from product use, like litter collection, and bans on product price discounts.[9,13,14] The World Health Organization considers such policies "best buys" for preventing and controlling non-communicable diseases.[15]

Despite strong evidence for the effectiveness of price-related policies, the implementation of such policies has been limited, due in part to industry efforts to undermine public and political support.[16-18] Indeed, substantial portions of the public remain sceptical of tax interventions and they are often described as nanny state policies or government overreach.[19-23] Despite this scepticism, support for efforts to protect children and to give everyone a comparable opportunity of achieving a healthy life are broadly supported.[24] This disconnect between a lack of public support for price-related policy interventions, yet strong support for protecting people's health represents an opportunity to reframe the debate.

For peer review only - http://bmjopen.bmj.com/site/about/guidelines.xhtml

As such, framing can be an important tool in public health advocacy efforts to gain public and policy support for policies that address corporate determinants of health.[25,26] Prior research on framing has enumerated frames used to address health-harming products.[27,28] In addition to understanding the role of the policy and the message's framing, it is important to know if there are differences in support by the type of product. Differences in support by the product could inform advocacy campaigns – especially if advocates move from a product-by-product approach to an umbrella approach for cancer-causing products.

To this end, as part of a US National Cancer Institute and Cancer Research U.K. sponsored Knowledge Integration Sandpit,[29] we formed a bi-national, interdisciplinary team to explore the use of framing in cancer-prevention pricing policies. We hypothesized that **(Hyp_1)** messages about non-tax pricing policies would result in greater levels of support for pricing policies than messages about tax policies, **(Hyp_2)** pricing policies would be differentially supported based on different frames devised through a qualitative development process, and **(Hyp_3)** tobacco-related policies would be more supported than alcohol- or sugar-related policies.

METHODS

This factorial experimental design contained three stages and a sample drawn from the UK and US public. In stage one, we conducted qualitative interviews with nine adults from the UK and nine adults from the US between February and May 2018. In total, we held 18 interviews, nine in each country (three members of the public, three cancer policy advocates, and three cancer survivors). We purposively sampled for diversity by sociodemographic identity. Trained interviewers in Scotland and North Carolina conducted interviews in their respective countries using a semi-structured interview guide. Briefly, we asked participants about their

For peer review only - http://bmjopen.bmj.com/site/about/guidelines.xhtml

initial thoughts, advantages/disadvantages, and benefits/harms of four pricing policies (minimum prices, banning use of coupons or discounts, adding taxes, and mitigation fees). We then asked about level of support for policies targeting specific products: alcohol, tobacco, and sugar. Finally, we asked what arguments for a pricing policy would be persuasive. All interviews were professionally transcribed verbatim, and transcriptions checked by the original interviewer. Transcripts were entered into QSR International's NVivo 12 qualitative data analysis software for thematic analysis.[30] Two researchers read all transcripts in depth and carried out initial coding following the interview guide structure. We then inductively identified recurrent themes for each price policy and compared similarities and differences in views between price policies, products, and countries.

In stage two, we used the results from the formative qualitative interviews and the published literature[20,24,28,31] to develop nine potential frames and iteratively refined them for clarity and readability. We then pilot-tested the frames, which are available online in our data repository, with 106 UK and US adults in an online survey panel provided by Qualtrics Research Services, 30th October-1st November 2018. Participants reported how well they understood the potential frames on a 3-point scale (1=understood very well, 2=understood fairly well, 3=did not understand well), described each in their own words and ranked the nine frames from most to least compelling. To select the frames for our main experiment, we computed the self-reported mean comprehensibility and the average ranking for each frame, and double coded (on a 3-point scale) whether participants accurately described the frame. In this step we did not manipulate product or policies; all messages were about alcohol taxes.

In the third and final stage, we selected four frames for an experiment. The four frames (Table 1) were adapted to each of three products (alcohol, sugar, tobacco) and each of three

For peer review only - http://bmjopen.bmj.com/site/about/guidelines.xhtml

policies (taxes, minimum pricing, elimination of discounting). We created 36 messages specific to product, policy, and the four frames about how the policy intervention would (1) reduce strain to healthcare, (2) protect children, (3) prevent cancer, and (4) bring consumption of the product down. All messages are provided in our linked Dataverse repository.

Table 1. Example frames

Frame Number (short description)	Example frame for alcohol product and tax policy
F_1 (reduce strain on healthcare)	The problems caused by alcohol misuse, such as crime, injuries and addiction, are costly. Increasing taxes on alcohol will reduce the financial strain on our overburdened health care system.
F_2 (protect children)	Alcohol misuse can hurt children's health and wellbeing - both directly (through children drinking alcohol), and indirectly (through adults' misuse of alcohol impacting children). We can protect children by increasing taxes on alcohol.
F_3 (prevent cancer)	Drinking less alcohol can reduce the risk of cancer. Increasing taxes on alcohol is an important way to prevent cancer in our communities.
F_4 (bring consumption down)	Drinking less alcohol can reduce the risk of stroke, high blood pressure, obesity, liver disease, and mental health problems. Increasing taxes on alcohol is an important way to bring drinking levels down.

Experimental design and implementation

We conducted an *a-priori* power analysis for the 3x4x3 factorial design using G*Power 3.1[32] with power (1 - β) set at 0.80 and α=.05, two-tailed, for a small effect size (Cohen's f =0.10) given prior findings examining similar constructs.[33,34] The power analysis indicated that a sample of 1745 would be needed to detect these small effects. To recruit participants, we engaged Qualtrics Research Services, an online panel service; this approach shows evidence of generalisability for experiments.[35,36] We used quota sampling to include 50% of participants from the UK and 50% from the US, equal representation by assigned birth sex, a minimum of 20% of participants with fewer than four years of post-secondary education, and a minimum of 20% of participants reporting having smoked 100 or more cigarettes in one's life. Qualtrics provided each participant "points" in exchange for completing the survey that could be redeemed

For peer review only - http://bmjopen.bmj.com/site/about/guidelines.xhtml

for incentives such as gift cards. Qualtrics successfully recruited and provided data from 1,850 participants. In this between-subjects experimental factorial design, each participant was randomly assigned to view one of the 36 messages using Qualtrics's advanced block programming. Figure 1 shows the experiment design with blue text for the cancer product and red text for the policy.

Figure 1. Frame x products x policy factorial design

		**Policy**		
		Tax	Discounting Ban	Min. Price
**Cancer Product**	**A**lcohol	F_1 A T	F_1 A D	F_1 A P
		F_2 A T	F_2 A D	F_2 A P
		F_3 A T	F_3 A D	F_3 A P
		F_4 A T	F_4 A D	F_4 A P
	**S**ugar	F_1 S T	F_1 S D	F_1 S P
		F_2 S T	F_2 S D	F_2 S P
		F_3 S T	F_3 S D	F_3 S P
		F_4 S T	F_4 S D	F_4 S P
	**T**obacco	F_1 T T	F_1 T D	F_1 T P
		F_2 T T	F_2 T D	F_2 T P
		F_3 T T	F_3 T D	F_3 T P
		F_4 T T	F_4 T D	F_4 T P

After brief screening and demographic questions, we provided the following prompt: "On the next screen, we will show you a message about a public health policy and a product. After you read the message, we'll ask you questions about what you think about policies about that product. PLEASE READ THE MESSAGE ABOUT THE PRODUCT ON THE NEXT SCREEN. You will not be able to see it again." The participant then viewed one of the 36 messages.

Measures

To assess the impact of the different products, policies, and frames in our messages, we used four dependent variables. Following prior work by Niederdeppe,[34] we first measured policy

For peer review only

support with three questions: "How much do you oppose or support the following?" i) Increasing taxes on the product mentioned in the message, ii) Getting rid of price discounts and special offers on the product mentioned in the message, and iii) Getting rid of low-cost versions of the product mentioned in the message. There were five response options ranging from Strongly Oppose (1) to Strongly Support (5) with a neutral point. Each participant answered all three questions regardless of the policy invoked in their message frame. We also measured reactance, a psychological response to having one's freedom limited,[37] to the message using six items adapted from Hall,[38] e.g., "The product mentioned is legal, so the government should stop interfering with it." There were five response options ranging from Strongly Disagree (1) to Strongly Agree (5) with a neutral point, which we averaged into a scale. These items exhibited good internal consistency in our data (McDonald's omega=0.87) and could not be improved by removing any item.[39]

We also measured potential covariates and factors including country (UK, US), and use of alcohol, sugary beverages, and cigarettes in the last 30 days (options: every day, some days, not at all). Full details of measures are available in our repository.

Analysis of Experiment

We assessed whether the: (1) type of policy, (2) product, or (3) frame used in a message was associated support for each type of policy and reactance, as well as interactions among these main predictors. We also assessed interactions between covariates (sex, age, country) and main predictors; if they were significant, those covariates were analysed as an additional factor as was the case for country in two of the models. All main effects and interactions were analysed using a 3x4x3 ANOVA. In two of the models, where country was significant, a 2x3x4x3 ANOVA was conducted. When main effects or interactions were significant, we conducted pairwise

For peer review only - http://bmjopen.bmj.com/site/about/guidelines.xhtml

comparisons using Fisher's least significant differences test. We used a traditional threshold of statistical significance, $p<0.05$ and used two tailed tests. We used SPSS v.25 (IBM, Chicago, IL).

Research undertaken in this project was reviewed by the East Carolina University and Medical Center Institutional Review Board (#17-002904) and University of Glasgow College of Medical, Veterinary & Life Sciences Ethics Committee (#200170074). Patients and the public were not involved in this research.

RESULTS

Formative interviews

We completed formative interviews with eighteen participants, nine in the UK and nine in the US. Interviewees were between ages 24 and 69 years (average age 48), and cancer survivors had a range of cancer experiences including prostate, lung, breast, head and neck, and skin cancers. Interviews lasted between twenty minutes and one hour depending on the interviewees' interest in the subject area. In general, interviewees were more supportive of price policies for tobacco containing commodities than either alcohol or sugar (Table 2).

Table 2: Interviewees' overall support for price policies in general by product type, country, and interviewee type, N=18

Product	UK interviewees									US interviewees									Mean
	Advocates			Survivors			Public			Advocates			Survivors			Public			
Tobacco	9	10	8	8	9	10	1	9	9	8	9	8	8	5	8	7	8	8	7.9
Alcohol	9	10	4	3	7	7	1	5	6	7	6	4	3	5	5	4	8	6	5.6
Sugar	10	10	3	8	4	8	3	4	9	5	3	2	7	5	2	5	6	4	5.4

Note: 1=not supportive, 10=very supportive.

During the interviews it became apparent that participants found it difficult to differentiate between a hypothecated tax and a mitigation fee. The team discussed this issue and decided to drop mitigation fee as a policy in the main experiment. A number of emergent themes were common to all three pricing policies (Table 3). Specifically, participants thought that all of

For peer review only - http://bmjopen.bmj.com/site/about/guidelines.xhtml

the policies would have an impact on purchasing behaviour with a concomitant health benefit due to reduced consumption, particularly for young people and those on low income. Participants also thought they could have an indirect benefit by drawing attention to the product and the level of potential health harm. For example, a policy advocate and cancer survivor said, respectively:

> '*…it would reduce the excessive consumption of these products, it would control the quantities in which these products are consumed, and it would mean the consumer… is more aware of their purchasing of the products.*' (Policy advocate)

> *'The biggest benefit is that it will make the person aware – some people drink and eat things they're not aware of, they don't look into it – and if they see that there's a higher tax, and they start recognising that, they're going to find out why and it might change their behaviour.'* (Cancer survivor)

[Table 3 appears on next page.]

For peer review only

Table 3. Themes identified in qualitative interviews

Common for all 3 pricing policies	Specific to minimum price	Specific to banning discounts	Specific to tax
**Supportive arguments/ advantages**			
• Health benefit<br>• Greatest benefit for young<br>• Greatest benefit for low SES<br>• Will affect purchasing behaviour<br>• Will highlight real cost of consumption/health harms<br>• More supportive where there is clear evidence of cancer links	• A deterrent or nudge<br>• Consistency and fairness<br>• Targets most harmful consumption<br>• There is evidence to support it	• Price promotions most strongly influence purchasing<br>• Promotions encourage over consumption<br>• Will protect industry and retailer profits	• Could prompt reformulation<br>• Could be a form of health insurance<br>• Source of public revenue to fund social policy programmes/ health services<br>• An effective way of moderating behaviour
**Unsupportive arguments/ disadvantages**			
• Effectiveness will depend on individual response<br>• Needs to be prohibitively high to change consumption<br>• Unfair on responsible consumers<br>• Addicts will pay more<br>• Could create a black market (US specific)<br>• Regressive<br>• Bad for retailers/ producers<br>• Simplistic – not enough	• Contravenes EU law<br>• Public do not understand/ will not support it<br>• Revenue raised goes to industry<br>• Too specific for food<br>• Will not impact population level drinking	• Lost opportunity to apply to healthy foods<br>• Will push prices up across the board<br>• Inappropriate to interfere in pricing	• Nanny statist - interference in freedom of choice<br>• Political cost – unpopular<br>• Reformulation could prompt increased consumption<br>• Negative unintended consequences - substitutes may be equally unhealthy<br>• Will not work if funds are not ring-fenced

For peer review only - http://bmjopen.bmj.com/site/about/guidelines.xhtml

Some key disadvantages were also highlighted across all policies: (1) that their effectiveness would depend on the scale of the price increase and how individuals responded; (2) that they are potentially regressive; and (3) unfair to responsible consumers and to those who are dependent on these substances. Participants were especially concerned about the impact on individuals dependent on these products with limited resources, particularly in relation to alcohol consumption. Yet, this concern also applied to tobacco and sugar, which were both deemed to have addictive qualities. Quotes illustrating these disadvantages include:

'The effect on health does depend on what decisions people make in response to it. And whether it follows what we expect. And does it reduce purchase of those products, does it push people to other products or not?' (Policy advocate)

'I think they [people with limited incomes] would then be more likely to stop buying food, to stop heating their house, or to stop paying their rent or, you know, whatever, in order to prioritize the alcohol consumption.' (General public)

There were a number of unique themes arising for each price policy (Table 3). For example, although discounts and price promotions were thought to be the strongest influencer of purchasing behaviour and to encourage over consumption, interviewees thought they should be refocused on healthier foods and drinks rather than banned. For minimum unit price policies specifically, interviewees noted that such policies target the most harmful patterns of consumption, i.e., the highest levels of consumption which can result in anti-social behaviour. Taxation alone prompted interviewees to talk about nanny state arguments and inappropriate government intervention in the market. This response was more marked in the UK than the US

For peer review only - http://bmjopen.bmj.com/site/about/guidelines.xhtml

and might be explained by the fact that the timing of the interviews coincided with the implementation of the UK Soft Drinks Industry Levy ("Sugar Tax") in April 2018.

The frames that interviewees thought would be most persuasive and result in increased support for price policies could be summarised as those that: (1) draw a very clear link to potential health harm from consuming the product; (2) focus on a positive health impact arising from the policy, particularly protection of children; (3) emphasise the impact on health services in terms of reduced costs and the potential to generate revenue for reinvestment; and, (4) make the policies real and relevant for people. Less convincing frames were those that: (1) employ threats or scare tactics; (2) seem condescending or nanny statist; (3) use jargon or statistics; and, (4) set unrealistic expectations of behaviour change for individuals. We used these findings to inform the experimental stimuli presented next.

Main experimental effects

Participants were 1,850 adults (ages 18-86, M_{age}=38.56, SD=13.56). The sample was evenly split across the countries of interest (50.1% from the UK) and biological sex (50.2% female). Within the sample, 34.7% graduated from college or university, 26.4% attended some college or university, 28.9% had graduated or completed secondary or high school, and the remainder (7.6%) attended some high school or less. Table 4 shows the pattern of results.

[Table 4 appears on the next page.]

For peer review only - http://bmjopen.bmj.com/site/about/guidelines.xhtml

Table 4. Summary of results, 2019, N=1,850, UK and US adults

Factors	Dependent variables			
	Support: Raising taxes	Support: Getting rid of price discounts and special offers	Support: Getting rid of low-cost version of product	Psychological Reactance
Policy	NS	NS	NS	NS
Frame	NS	• Higher support with Frame 2 (Children) and 3 (Prevent Cancer) than 4 (Reduce Use)	• For participants receiving Frame 3 (Prevent Cancer) support was higher for minimum pricing policy in message than for tax policy in message<br>• For participants receiving Frame 4 (Reduce Use) differences in support by policy in message	• Frame 2 (Children) lowered reactance for UK participants more than US participants
Product	•Tobacco: Highest<br>•Alcohol: Lower<br>•Sugar: Lowest<br>•Tobacco higher for UK than US	•Tobacco: Highest<br>•Alcohol: Lower<br>•Sugar: Lowest	•Tobacco: Highest<br>•Alcohol: Lower<br>•Sugar: Lowest	•Sugar: Highest<br>•Alcohol: Lower<br>•Tobacco: Lowest<br>• Sugar and Tobacco lower for UK than US
Country†	•UK: Higher<br>•US: Lower	NS	NS	•US: Higher<br>•UK: Lower

NS=not statistically significant; † Covariate assessed as a factor given significant interactions with other factors

Outcome 1: Support for increasing taxes on the product mentioned in the message

For the first outcome, there was not a significant main effect for policy or frame. There was a significant main effect for message product (F[2, 1767]=45.71, p<0.001) and country (F[1, 1767]=26.63, p<0.001). Pairwise comparisons for product revealed significant differences across all three products (M_{sugar}=2.76, SD=1.26; $M_{alcohol}$=3.07, SD=1.30; $M_{tobacco}$=3.47; SD=1.37); participants receiving a message regarding sugar had the lowest and participants receiving a

For peer review only - http://bmjopen.bmj.com/site/about/guidelines.xhtml

message regarding tobacco endorsed the highest levels of support for increasing taxes on the product. Pairwise comparisons for country revealed that participants from the UK (M_{UK}=3.257, *SD*=1.33) endorsed higher levels of support for increasing taxes compared to participants from the US (M_{US}=2.937, *SD*=1.34). There was also a significant two-way interaction: product x country, $F(2, 1767)=5.67$, $p<0.01$. Specifically, among participants receiving a sugar product message, those from the UK endorsed higher levels of support for increasing taxes on the product (M_{UK}=3.08, *SD*=1.28) compared to US participants (M_{US}=2.51, *SD*=1.19). Similarly, among participants receiving a tobacco product message, those from the UK endorsed higher levels of support for increasing taxes on the product (M_{UK}=3.69, *SD*=1.32) compared to US participants (M_{US}=3.24, *SD*=1.38). Three-way interactions were not significant.

Outcome 2: Support for getting rid of price discounts and special offers on the product mentioned in the message

For the second outcome, there was not a significant main effect for policy. There was a significant main effect for frame ($F[3, 1803]=2.62$, $p<0.05$) and message product ($F[2, 1803]=53.80$, $p<0.001$). Participants receiving frames 2 (protect children, M_{frame2}=3.25, *SD*=1.37) or 3 (prevent cancer, M_{frame3}=3.24, *SD*=1.35) endorsed higher levels of support for getting rid of price discounts and special offers than participants who received frame 4 (bring consumption down, M_{frame4}=3.04, *SD*=1.36). Pairwise comparisons for product revealed significant differences across all three products ($M_{sugar=}$2.85, *SD*=1.28; $M_{alcohol}$=3.05, SD=1.34; $M_{tobacco}$=3.59; *SD*=1.35); participants receiving a message regarding sugar and participants receiving a message regarding tobacco endorsed the lowest and highest levels of support for getting rid of price discounts and special offers on the product, respectively. There were no significant two-way or three-way interactions.

For peer review only

For peer review only - http://bmjopen.bmj.com/site/about/guidelines.xhtml

350 **Outcome 3: Support for getting rid of low-cost versions of the product mentioned in the**
351 **message**

352 For the third outcome, there was not a significant main effect for policy or frame. There
353 was a significant main effect for message product (*F*(2, 1802)=60.34, $p<0.001$). Pairwise
354 comparisons revealed significant differences across all three products ($M_{sugar=}$2.82, *SD*=1.26;
355 $M_{alcohol}$=3.08, *SD*=1.29; $M_{tobacco}$=3.60; *SD*=1.34), such that participants receiving a message
356 regarding sugar and participants receiving a message regarding tobacco endorsed the lowest and
357 highest levels of support for getting rid of low-cost versions of the product, respectively. There
358 was also a significant two-way interaction: frame x policy, F(6, 1802)=2.24, p<0.05.
359 Specifically, among participants receiving frame 3 (prevent cancer), participants who received
360 the policy related to minimum pricing endorsed higher levels of support for getting rid of low-
361 cost versions of the product ($M_{minprice}$=3.361, *SD*=1.28) compared to those receiving the policy
362 related to increasing taxes (M_{tax}=3.054, *SD*=1.38). Additionally, among participants receiving
363 frame 4 (bring consumption down), there were significant differences in support for getting rid
364 of low-cost products between participants who received policies related to discounting bans
365 ($M_{discountingban}$=3.226, *SD*=1.30) and minimum pricing ($M_{minprice}$=3.164, *SD*=1.37), and
366 participants who received the policy related to increasing taxes (M_{tax}=2.764, *SD*=1.29). There
367 were no significant three-way interactions.

368 **Outcome 4: Reactance**

369 For the last outcome, there was not a significant main effect for policy or frame. There
370 was a significant main effect for message product (*F*[2, 1769]=30.74, $p<0.001$) and country
371 (*F*[1, 1769]=7.21, $p<0.01$). Pairwise comparisons for product revealed significant differences
372 across all three products ($M_{tobacco}$=2.72; *SD*=0.99; $M_{alcohol}$=2.92, *SD*=0.95; $M_{sugar=}$3.14,

For peer review only - http://bmjopen.bmj.com/site/about/guidelines.xhtml

SD=0.93), such that participants receiving a message regarding tobacco and participants receiving a message regarding sugar, endorsed the weakest and strongest levels of agreement on items from the reactance scale, respectively. Pairwise comparisons for country revealed that participants from the US (M_{US}=2.99, *SD*=0.97) endorsed stronger levels of agreement on items from the reactance scale compared to participants from the UK (M_{UK}=2.87, *SD*=0.97). There were two significant two-way interactions: product x country, $F(2, 1769)=4.09$, $p<0.05$ and frame x country $F(3, 1769)=2.84$, p<0.05. Specifically, among those that received a message regarding tobacco, participants from the US endorsed significantly stronger levels of agreement (M_{US}=2.838, *SD*=0.98) compared to participants from the UK (M_{UK}=2.606, *SD*=0.98). Similarly, among those receiving a message regarding sugar, participants from the US endorsed significantly stronger levels of agreement (M_{US}=3.24, *SD*=0.91) compared to participants from the UK (M_{UK}=3.05, *SD*=0.93). Among those who received frame 2 (protect children), participants from the UK endorsed weaker agreement (M_{UK}=2.71, *SD*=0.97) on the reactance scale than those from the US (M_{US}=3.01, *SD*=0.93).

Lastly, there was also a significant three-way interaction: product x policy x country, ($F[4, 1769]=2.97$, $p<0.05$). Among those receiving a message regarding alcohol, those from the UK endorsed significantly stronger levels of agreement (M_{UK}=3.05, *SD*=0.92) compared to participants from the US (M_{US}=2.78, SD=0.94) receiving the discounting ban policy. In contrast, among those receiving a message regarding sugar, those from the US endorsed significantly stronger levels of agreement (M_{US}=3.39, *SD*=0.92) compared to participants from the UK (M_{UK}=3.07, *SD*=0.95) for those receiving the policy related to increasing taxes. Among those receiving a message regarding tobacco, those from the US endorsed significantly stronger levels

For peer review only

For peer review only - http://bmjopen.bmj.com/site/about/guidelines.xhtml

395 of agreement (M_{US}=2.93, SD=1.05) compared to participants from the UK (M_{UK}=2.48, SD=0.95)
396 receiving the discounting ban policy.

397 **DISCUSSION**

398 **Principal findings**

399 This research aimed to explore whether the specific content of a message about cancer-
400 prevention price policies was associated with higher public support and lower reactance for those
401 policies. We had three main hypotheses. First, we hypothesized that messages about non-tax
402 pricing policies would result in more support than messages about tax policies. Our results did
403 not provide evidence for this hypothesis: Not one of the three policies tested (taxes, minimum
404 pricing, and bans on discounts) showed a statistically significant impact on support in our
405 experiment. Regarding our second hypothesis, that support for a particular pricing policy would
406 change based on the framing of the message, we found some evidence. Specifically, our findings
407 indicate that frames relating to children and those relating to reducing risk of cancer may provide
408 the most promise. We found evidence to support our third hypothesis that messages about
409 tobacco products would result in more support for raising the price of products than messages
410 about alcohol or sugar products. Product in the message also changed reactance, which was
411 highest for sugar and lowest for tobacco. Finally, we found statistically significant differences in
412 responses to messages by country (e.g., support for alcohol pricing interventions was lower in
413 the US than in the UK).

414 **Strengths and weaknesses**

415 The strong internal validity from the experimental design, qualitative formative work,
416 and international scope of this research must be balanced against limitations. First, this study
417 used an online convenience sample from a panel provider service, Qualtrics, which may limit its

For peer review only

external validity. However, prior research indicates that convenience samples like ours generalize well when used in experimental studies rather than studies of prevalence.[35] Second, we assessed the impact of a single exposure of a message displayed on a screen. This does not reflect the real-world environment where messages about policies may be delivered from multiple sources, repeated, and seen on multiple communication channels.

Results in context

The process of policy development and policy making decisions are ultimately taken by politicians on behalf of their constituents (the public), where the scientific evidence- and value-bases play important roles.[40] While the evidence-base for different types and targets of price policies on behaviour is rapidly expanding,[41-46] the difficulty of increasing public support and consequently political support for pricing interventions has received less attention. The relatively low levels of public support have previously been highlighted.[20,47] Our findings provide further evidence to suggest limited support among the public for pricing interventions overall, given relatively low endorsements of support on our support dependent variables. However, given the mean scores, the average participant was also not that opposed. Furthermore, a policy message that specifically referenced a particular type of price policy did not result in more support for that type of price policy compared to other types. Prior research has examined the role of framing in public health efforts, and our findings demonstrate ways to best utilize media advocacy approaches[48] by minimizing reactance from the public and promoting support of evidence-based policies. Specifically, our frames were all selected based on the prior literature and their performance in formative work. Particular value may come from framing messages around the protection of children and reduction of cancer risk. Previous work has shown that the product under consideration influences policy support.[49] Our experimental findings offer further evidence

For peer review only - http://bmjopen.bmj.com/site/about/guidelines.xhtml

of this, showing that participants rated support of pricing interventions highest on messages for tobacco products, followed by alcohol, and lastly by sugar. Finally, our findings highlight some notable differences by country, which may reflect more general differences in acceptance of policies, healthcare systems, and political developments at the time of our study (e.g., 'Brexit').

Conclusions

Our findings have several implications for improving the framing pricing policies and for policy research. First, the evidence of differences by product indicate that success of tobacco denormalization efforts may have yet to translate to alcohol and sugar efforts. It is worth highlighting that denormalization can present the risk of increasing stigma, which has been a criticism in the area of tobacco.[50] Second, our findings suggest the importance of bridging the research-practice gap between the literature on framing and advocacy organizations' messaging. Particularly useful frames may include a focus on cancer prevention and protecting children. Third, advocates and researchers should know that the messages may work across different types of pricing policies. Fourth, differences by country may help with identifying particular challenges and successes regarding cancer prevention that can then be leveraged elsewhere. Finally, our findings can inform population health interventions[51] and underscore the need for research that accelerates the adoption and implementation of evidence-based policy interventions. The results presented herein highlight the importance of connecting research on human behaviour and message framing with cancer control advocates and non-governmental organizations working to advance evidence-based policies. Our approach with a diverse group of expertise and formative research, may serve as a model for how to connect behavioural science with cancer prevention policy advocacy efforts. Future work extending ours is critical to

For peer review only - http://bmjopen.bmj.com/site/about/guidelines.xhtml

advancing the adoption of evidence-based population-level interventions to prevent cancer-related morbidity and mortality.

Author Contributions

JGLL, RNC, EMT, DIC conceived the study. JGLL, CHB, RNC, EMT, TI, SH, SDG, and DIC designed the study. JVC (quantitative) and CHB (qualitative) conducted the data analysis with input from EMT and SH, respectively. PMS conducted analyses of the pilot study with input from RNC. JGLL, CHB, RNC, EMT, PMS, SDG, and DIC developed the stimuli. All authors provided critical input on the interpretation of the results, writing of the manuscript, and approved the final version.

Human Participant Protections

Research undertaken in this project was reviewed by the East Carolina University and Medical Center Institutional Review Board (#17-002904) and University of Glasgow College of Medical, Veterinary & Life Sciences Ethics Administration Committee (#200170074).

Acknowledgements

We thank Cancer Research UK and the US National Cancer Institute Health Behaviours Research Branch for the opportunity to participate in their jointly hosted 2017 “sandpit” workshop. We thank Israel M. Mendez for his help with the data collection and project management in the US. An earlier version of this paper was presented in a dissemination event held at the Royal Institution in London in November 2019.

For peer review only

For peer review only - http://bmjopen.bmj.com/site/about/guidelines.xhtml

REFERENCES

1 Parkin, D. M., Boyd, L. & Walker, L. C. 16. The fraction of cancer attributable to lifestyle and environmental factors in the UK in 2010. *Br. J. Cancer* **105 Suppl 2**, S77-81, doi:10.1038/bjc.2011.489 (2011).

2 Madureira Lima, J. & Galea, S. Corporate practices and health: a framework and mechanisms. *Global Health* **14**, 21, doi:10.1186/s12992-018-0336-y (2018).

3 McKee, M. & Stuckler, D. Responding to the Corporate and Commercial Determinants of Health. *Am J Public Health*, e1-e4, doi:10.2105/ajph.2018.304510 (2018).

4 Contreary, K. A. *et al.* Economic Impact of Tobacco Price Increases Through Taxation: A Community Guide Systematic Review. *Am J Prev Med* **49**, 800-808, doi:10.1016/j.amepre.2015.04.026 (2015).

5 IARC. in *IARC handbooks of cancer prevention* Vol. 14 (International Agency for Research on Cancer, Lyon, France, 2011).

6 Wagenaar, A. C., Salois, M. J. & Komro, K. A. Effects of beverage alcohol price and tax levels on drinking: a meta-analysis of 1003 estimates from 112 studies. *Addiction* **104**, 179-190, doi:10.1111/j.1360-0443.2008.02438.x (2009).

7 Powell, L. M., Chriqui, J. F., Khan, T., Wada, R. & Chaloupka, F. J. Assessing the potential effectiveness of food and beverage taxes and subsidies for improving public health: a systematic review of prices, demand and body weight outcomes. *Obesity reviews : an official journal of the International Association for the Study of Obesity* **14**, 110-128, doi:10.1111/obr.12002 (2013).

For peer review only

For peer review only - http://bmjopen.bmj.com/site/about/guidelines.xhtml

8 Burton, R. *et al.* A rapid evidence review of the effectiveness and cost-effectiveness of alcohol control policies: an English perspective. *Lancet* **389**, 1558-1580, doi:10.1016/s0140-6736(16)32420-5 (2017).

9 Boniface, S., Scannell, J. W. & Marlow, S. Evidence for the effectiveness of minimum pricing of alcohol: a systematic review and assessment using the Bradford Hill criteria for causality. *BMJ Open* **7**, e013497, doi:10.1136/bmjopen-2016-013497 (2017).

10 Eyles, H., Ni Mhurchu, C., Nghiem, N. & Blakely, T. Food pricing strategies, population diets, and non-communicable disease: a systematic review of simulation studies. *PLoS Med* **9**, e1001353, doi:10.1371/journal.pmed.1001353 (2012).

11 Afshin, A. *et al.* The prospective impact of food pricing on improving dietary consumption: A systematic review and meta-analysis. *PLoS One* **12**, e0172277, doi:10.1371/journal.pone.0172277 (2017).

12 Teng, A. M. *et al.* Impact of sugar-sweetened beverage taxes on purchases and dietary intake: Systematic review and meta-analysis. *Obesity reviews : an official journal of the International Association for the Study of Obesity* **20**, 1187-1204, doi:10.1111/obr.12868 (2019).

13 Golden, S. D., Farrelly, M. C., Luke, D. A. & Ribisl, K. M. Comparing projected impacts of cigarette floor price and excise tax policies on socioeconomic disparities in smoking. *Tob Control* **25**, i60-i66, doi:10.1136/tobaccocontrol-2016-053230 (2016).

14 Golden, S. D. *et al.* Beyond excise taxes: a systematic review of literature on non-tax policy approaches to raising tobacco product prices. *Tob Control* **25**, 377-385, doi:10.1136/tobaccocontrol-2015-052294 (2016).

For peer review only

For peer review only - http://bmjopen.bmj.com/site/about/guidelines.xhtml

15 World Health Organization. *Tackling NCDs: 'Best buys' and other recommended interventions for the prevention and control of noncommunicable diseases*, <https://www.who.int/ncds/management/best-buys/en/> (2017).

16 Berg, C. J. *et al.* Tobacco Taxes in the Southeastern US States: Views from Former Legislators. *Health behavior and policy review* **2**, 333-342, doi:10.14485/hbpr.2.5.1 (2015).

17 Katikireddi, S. V. & Hilton, S. How did policy actors use mass media to influence the Scottish alcohol minimum unit pricing debate? Comparative analysis of newspapers, evidence submissions and interviews. *Drugs (Abingdon, England)* **22**, 125-134, doi:10.3109/09687637.2014.977228 (2015).

18 Holmes, C. B., King, B. A. & Babb, S. D. Stuck in Neutral: Stalled Progress in Statewide Comprehensive Smoke-Free Laws and Cigarette Excise Taxes, United States, 2000-2014. *Prev Chronic Dis* **13**, E80, doi:10.5888/pcd13.150409 (2016).

19 Stockwell, T. Minimum unit pricing for alcohol: the most cost-effective of cancer prevention strategies? *Expert review of anticancer therapy* **17**, 981-983, doi:10.1080/14737140.2017.1381565 (2017).

20 Barry, C. L., Niederdeppe, J. & Gollust, S. E. Taxes on sugar-sweetened beverages: results from a 2011 national public opinion survey. *Am J Prev Med* **44**, 158-163, doi:10.1016/j.amepre.2012.09.065 (2013).

21 Petrescu, D. C., Hollands, G. J., Couturier, D. L., Ng, Y. L. & Marteau, T. M. Public Acceptability in the UK and USA of Nudging to Reduce Obesity: The Example of Reducing Sugar-Sweetened Beverages Consumption. *PLoS One* **11**, e0155995, doi:10.1371/journal.pone.0155995 (2016).

For peer review only

22 Eykelenboom, M. *et al.* Political and public acceptability of a sugar-sweetened beverages tax: a mixed-method systematic review and meta-analysis. *Int J Behav Nutr Phys Act* **16**, 78, doi:10.1186/s12966-019-0843-0 (2019).

23 Weishaar, H. *et al.* Why media representations of corporations matter for public health policy: a scoping review. *BMC Public Health* **16**, 899, doi:10.1186/s12889-016-3594-8 (2016).

24 RWJF. *A new way to talk about the social determinants of health*, <http://www.rwjf.org/content/dam/farm/reports/reports/2010/rwjf63023> (2010).

25 Jacoby, W. G. Issue framing and public opinion on government spending. *American Journal of Political Science*, 750-767 (2000).

26 Wallack, L. & Dorfman, L. Media advocacy: a strategy for advancing policy and promoting health. *Health Educ Q* **23**, 293-317 (1996).

27 Roth, A. L., Dunsby, J. & Bero, L. A. Framing processes in public commentary on US federal tobacco control regulation. *Social Studies of Science* **33**, 7-44 (2003).

28 Niederdeppe, J., Gollust, S. E., Jarlenski, M. P., Nathanson, A. M. & Barry, C. L. News coverage of sugar-sweetened beverage taxes: pro- and antitax arguments in public discourse. *Am J Public Health* **103**, e92-98, doi:10.2105/ajph.2012.301023 (2013).

29 National Cancer Institute Behavioral Research Program. *NCI-CRUK Sandpit Workshops*, <https://cancercontrol.cancer.gov/brp/hbrb/sandpit.html> (2017).

30 Braun, V. & Clarke, V. in *APA handbook of research methods in psychology: Research designs: Quantitative, qualitative, neuropsychological, and biological* Vol. 2 (eds H. Cooper *et al.*) (American Psychological Association, 2012).

For peer review only

For peer review only - http://bmjopen.bmj.com/site/about/guidelines.xhtml

31 Katikireddi, S. V., Bond, L. & Hilton, S. Changing policy framing as a deliberate strategy for public health advocacy: a qualitative policy case study of minimum unit pricing of alcohol. *Milbank Q.* **92**, 250-283, doi:10.1111/1468-0009.12057 (2014).

32 Faul, F., Erdfelder, E., Lang, A. G. & Buchner, A. G*Power 3: a flexible statistical power analysis program for the social, behavioral, and biomedical sciences. *Behav Res Methods* **39**, 175-191 (2007).

33 Gollust, S. E., Niederdeppe, J. & Barry, C. L. Framing the consequences of childhood obesity to increase public support for obesity prevention policy. *Am J Public Health* **103**, e96-102, doi:10.2105/ajph.2013.301271 (2013).

34 Niederdeppe, J., Roh, S. & Shapiro, M. A. Acknowledging individual responsibility while emphasizing social determinants in narratives to promote obesity-reducing public policy: a randomized experiment. *PLoS One* **10**, e0117565, doi:10.1371/journal.pone.0117565 (2015).

35 Jeong, M. *et al.* Similarities and Differences in Tobacco Control Research Findings From Convenience and Probability Samples. *nn. Behav. Med.* **53**, 476-485, doi:10.1093/abm/kay059 (2019).

36 Brandon, D. M., Long, J. H., Loraas, T. M., Mueller-Phillips, J. & Vansant, B. Online instrument delivery and participant recruitment services: Emerging opportunities for behavioral accounting research. *Behavioral Research in Accounting* **26**, 1-23 (2013).

37 Brehm, S. S. & Brehm, J. W. *Psychological reactance: A theory of freedom and control.* (Academic Press, 1981).

38 Hall, M. G. *et al.* Reactance to Health Warnings Scale: Development and Validation. *nn. Behav. Med.* **50**, 736-750, doi:10.1007/s12160-016-9799-3 (2016).

For peer review only

For peer review only - http://bmjopen.bmj.com/site/about/guidelines.xhtml

39 Hayes, A. F. *My Macros and Code for SPSS and SAS: OMEGA*, <http://afhayes.com/spss-sas-and-mplus-macros-and-code.html> (2019).

40 Oliver, K. & Cairney, P. The dos and don’ts of influencing policy: a systematic review of advice to academics. *Palgrave Communications* **5**, 21, doi:10.1057/s41599-019-0232-y (2019).

41 Wagenaar, A. C., Tobler, A. L. & Komro, K. A. Effects of alcohol tax and price policies on morbidity and mortality: a systematic review. *Am J Public Health* **100**, 2270-2278, doi:10.2105/ajph.2009.186007 (2010).

42 Bennett, R. *et al.* Prevalence of healthy and unhealthy food and beverage price promotions and their potential influence on shopper purchasing behaviour: A systematic review of the literature. *Obesity reviews : an official journal of the International Association for the Study of Obesity* **21**, e12948, doi:10.1111/obr.12948 (2020).

43 Pimpin, L., Sassi, F., Corbould, E., Friebel, R. & Webber, L. Fiscal and pricing policies to improve public health: a review of the evidence. (Public Health England, London, UK, 2018).

44 Martin, L., Bauld, L. & Angus, K. Rapid evidence review: The impact of promotions on high fat, sugar and salt (HFSS) food and drink on consumer purchasing and consumption behaviour and the effectiveness of retail environment interventions. (NHS Health Scotland, Edinburgh, 2017).

45 Whitehead, R. *et al.* Rapid evidence review: Strengths and limitations of tobacco taxation and pricing strategies. (NHS Health Scotland, Edinburgh, 2018).

46 World Health Organization Regional Office for Europe. *Using price policies to promote healthier diets*. (WHO Regional Office for Europe, 2015).

For peer review only

For peer review only - http://bmjopen.bmj.com/site/about/guidelines.xhtml

47 Dorfman, L. Talking about sugar sweetened-beverage taxes: will actions speak louder than words? *Am J Prev Med* **44**, 194-195, doi:10.1016/j.amepre.2012.11.002 (2013).

48 Dorfman, L. & Krasnow, I. D. Public health and media advocacy. *Annu Rev Public Health* **35**, 293-306, doi:10.1146/annurev-publhealth-032013-182503 (2014).

49 Diepeveen, S., Ling, T., Suhrcke, M., Roland, M. & Marteau, T. M. Public acceptability of government intervention to change health-related behaviours: a systematic review and narrative synthesis. *BMC Public Health* **13**, 756, doi:10.1186/1471-2458-13-756 (2013).

50 Frohlich, K. L., Mykhalovskiy, E., Poland, B. D., Haines-Saah, R. & Johnson, J. Creating the socially marginalised youth smoker: the role of tobacco control. *Sociol. Health Illn.* **34**, 978-993, doi:10.1111/j.1467-9566.2011.01449.x (2012).

51 Hawe, P. & Potvin, L. What is population health intervention research? *Can J Public Health* **100**, Suppl I8-14 (2009).

For peer review only

For peer review only - http://bmjopen.bmj.com/site/about/guidelines.xhtml

STROBE 2007 (v4) Statement—Checklist of items that should be included in reports of *cross-sectional studies*

As there is no applicable checklist for a factorial design, we are utilizing the STROBE statement.

Section/Topic	Item #	Recommendation	Reported on page #
**Title and abstract**	1	(*a*) Indicate the study's design with a commonly used term in the title or the abstract	1
		(*b*) Provide in the abstract an informative and balanced summary of what was done and what was found	2
**Introduction**			
Background/rationale	2	Explain the scientific background and rationale for the investigation being reported	4-5
Objectives	3	State specific objectives, including any prespecified hypotheses	5
**Methods**			
Study design	4	Present key elements of study design early in the paper	5
Setting	5	Describe the setting, locations, and relevant dates, including periods of recruitment, exposure, follow-up, and data collection	5-8
Participants	6	(*a*) Give the eligibility criteria, and the sources and methods of selection of participants	7-8
Variables	7	Clearly define all outcomes, exposures, predictors, potential confounders, and effect modifiers. Give diagnostic criteria, if applicable	8-9
Data sources/ measurement	8*	For each variable of interest, give sources of data and details of methods of assessment (measurement). Describe comparability of assessment methods if there is more than one group	8-9 (+ codebook in repository)
Bias	9	Describe any efforts to address potential sources of bias	9
Study size	10	Explain how the study size was arrived at	7
Quantitative variables	11	Explain how quantitative variables were handled in the analyses. If applicable, describe which groupings were chosen and why	9-10
Statistical methods	12	(*a*) Describe all statistical methods, including those used to control for confounding	9-10
		(*b*) Describe any methods used to examine subgroups and interactions	9-10
		(*c*) Explain how missing data were addressed	N/A – minimal sporadic missingness

For peer review only

		(*d*) If applicable, describe analytical methods taking account of sampling strategy	N/A
		(*e*) Describe any sensitivity analyses	N/A
**Results**			
Participants	13*	(a) Report numbers of individuals at each stage of study—eg numbers potentially eligible, examined for eligibility, confirmed eligible, included in the study, completing follow-up, and analysed	N/A
		(b) Give reasons for non-participation at each stage	N/A
		(c) Consider use of a flow diagram	N/A
Descriptive data	14*	(a) Give characteristics of study participants (eg demographic, clinical, social) and information on exposures and potential confounders	14
		(b) Indicate number of participants with missing data for each variable of interest	N/A – Minimal sporadic missingness
Outcome data	15*	Report numbers of outcome events or summary measures	15-18
Main results	16	(*a*) Give unadjusted estimates and, if applicable, confounder-adjusted estimates and their precision (eg, 95% confidence interval). Make clear which confounders were adjusted for and why they were included	15-18
		(*b*) Report category boundaries when continuous variables were categorized	Codebook
		(*c*) If relevant, consider translating estimates of relative risk into absolute risk for a meaningful time period	N/A
Other analyses	17	Report other analyses done—eg analyses of subgroups and interactions, and sensitivity analyses	N/A
**Discussion**			
Key results	18	Summarise key results with reference to study objectives	19
Limitations	19	Discuss limitations of the study, taking into account sources of potential bias or imprecision. Discuss both direction and magnitude of any potential bias	19-20
Interpretation	20	Give a cautious overall interpretation of results considering objectives, limitations, multiplicity of analyses, results from similar studies, and other relevant evidence	20-21
Generalisability	21	Discuss the generalisability (external validity) of the study results	19-20
**Other information**			
Funding	22	Give the source of funding and the role of the funders for the present study and, if applicable, for the original study on which the present article is based	3

*Give information separately for cases and controls in case-control studies and, if applicable, for exposed and unexposed groups in cohort and cross-sectional studies.

For peer review only

Note: An Explanation and Elaboration article discusses each checklist item and gives methodological background and published examples of transparent reporting. The STROBE checklist is best used in conjunction with this article (freely available on the Web sites of PLoS Medicine at http://www.plosmedicine.org/, Annals of Internal Medicine at http://www.annals.org/, and Epidemiology at http://www.epidem.com/). Information on the STROBE Initiative is available at www.strobe-statement.org.

For peer review only

BMJ Open

Message framing to inform cancer prevention pricing interventions in the UK and US: A factorial experiment, 2019

Journal:	*BMJ Open*
Manuscript ID	bmjopen-2020-041324.R1
Article Type:	Original research
Date Submitted by the Author:	10-Nov-2020
Complete List of Authors:	Lee, Joseph; East Carolina University, Department of Health Education and Promotion, College of Health and Human Performance; University of North Carolina Lineberger Comprehensive Cancer Center, Cancer Prevention and Control<br>Cristello, Julie; Florida International University,<br>Buckton, Christina; University of Glasgow, MRC/CSO Social and Public Health Sciences Unit, Institute of Health & Wellbeing<br>Carey, Rachel; University College London, Department of Clinical, Educational & Health Psychology<br>Trucco, Elisa; Florida International University, Department of Psychology and Center for Children and Families; University of Michigan, Department of Psychiatry<br>Schenk, Paulina; University College London, Department of Clinical, Educational & Health Psychology<br>Ikegwuonu, Theresa; University of Glasgow, MRC/CSO Social and Public Health Sciences Unit, Institute of Health & Wellbeing<br>Hilton , Shona; University of Glasgow, MRC/CSO Social and Public Health Sciences Unit, Institute of Health & Wellbeing<br>Golden, SD; University of North Carolina at Chapel Hill Gillings School of Global Public Health; University of North Carolina Lineberger Comprehensive Cancer Center, Cancer Prevention and Control<br>Conway, D; University of Glasgow, School of Medicine, Dentistry, and Nursing, College of Medical Veterinary and Life Sciences
**Primary Subject Heading**:	Public health
Secondary Subject Heading:	Communication, Health policy, Oncology
Keywords:	Health policy < HEALTH SERVICES ADMINISTRATION & MANAGEMENT, PREVENTIVE MEDICINE, PUBLIC HEALTH

SCHOLARONE™
Manuscripts

For peer review only - http://bmjopen.bmj.com/site/about/guidelines.xhtml

I, the Submitting Author has the right to grant and does grant on behalf of all authors of the Work (as defined in the below author licence), an exclusive licence and/or a non-exclusive licence for contributions from authors who are: i) UK Crown employees; ii) where BMJ has agreed a CC-BY licence shall apply, and/or iii) in accordance with the terms applicable for US Federal Government officers or employees acting as part of their official duties; on a worldwide, perpetual, irrevocable, royalty-free basis to BMJ Publishing Group Ltd ("BMJ") its licensees and where the relevant Journal is co-owned by BMJ to the co-owners of the Journal, to publish the Work in this journal and any other BMJ products and to exploit all rights, as set out in our licence.

The Submitting Author accepts and understands that any supply made under these terms is made by BMJ to the Submitting Author unless you are acting as an employee on behalf of your employer or a postgraduate student of an affiliated institution which is paying any applicable article publishing charge ("APC") for Open Access articles. Where the Submitting Author wishes to make the Work available on an Open Access basis (and intends to pay the relevant APC), the terms of reuse of such Open Access shall be governed by a Creative Commons licence – details of these licences and which Creative Commons licence will apply to this Work are set out in our licence referred to above.

Other than as permitted in any relevant BMJ Author's Self Archiving Policies, I confirm this Work has not been accepted for publication elsewhere, is not being considered for publication elsewhere and does not duplicate material already published. I confirm all authors consent to publication of this Work and authorise the granting of this licence.

For peer review only

Public Health

For submission to: *BMJ Open*

Message framing to inform cancer prevention pricing interventions in the UK and US: A factorial experiment, 2019

Joseph G. L. Lee, PhD, MPH,[1,2*] Julie V. Cristello, MS,[3,4] Christina H. Buckton, MSc, MBA,[5] Rachel N. Carey, PhD,[6] Elisa M. Trucco, PhD,[3,4,7] Paulina M. Schenk, MSc,[6] Theresa Ikegwuonu, PhD,[5] Prof. Shona Hilton, PhD,[5] Shelley D. Golden, PhD, MPH,[2,8] Prof. David I. Conway, BDS, FDS, MPH, FFPH, PhD[9]

1. Department of Health Education and Promotion, College of Health and Human Performance, East Carolina University, Greenville, NC, USA

2. Cancer Prevention and Control, UNC Lineberger Comprehensive Cancer Center, Chapel Hill, NC, USA

3. Department of Psychology, Florida International University, Miami, FL, USA

4. Center for Children and Families, Florida International University, Miami, FL, USA

5. MRC/CSO Social and Public Health Sciences Unit, Institute of Health & Wellbeing, University of Glasgow, Glasgow, UK

6. Department of Clinical, Educational & Health Psychology, University College London, London, UK

7. Department of Psychiatry, University of Michigan, Ann Arbor, MI, USA

8. Department of Health Behavior, UNC Gillings School of Global Public Health, Chapel Hill, NC, USA

9. School of Medicine, Dentistry, and Nursing, College of Medical Veterinary and Life Sciences, University of Glasgow, Glasgow, UK

* Corresponding author: Dr. Joseph G. L. Lee, Department of Health Education and Promotion, East Carolina University, 1000 East Fifth Street Stop 529, Greenville, NC 27858; phone: +1 (252) 328-4661; fax: +1 (252) 328-1285; leejose14@ecu.edu

Key Words: public policy, policy making, prevention and control, taxes, neoplasms
Word Count: 4,830

For peer review only - http://bmjopen.bmj.com/site/about/guidelines.xhtml

Abstract (293/300 words)

Objectives: To advance understanding of how message framing can be used to maximise public support across different pricing policies for alcohol, tobacco, and sugary drinks/foods that prevent consumption of cancer-causing products.

Design: We designed a 3x4x3 randomised factorial experiment to test responses to messages with three pricing policies, four message frames, and three products.

Setting: Online survey panel (Qualtrics) in 2019.

Participants: Adults (N=1850) from the UK and USA.

Interventions: Participants randomly viewed one of 36 separate messages that varied by pricing policy (increasing taxes, getting rid of price discounts, getting rid of low-cost products), four frames, and product (alcohol, tobacco, sugary drinks/foods).

Primary and secondary outcome measures: We assessed the relationship between the message characteristics and four dependent variables. Three were related to policy support: (1) increasing taxes on the product mentioned in the message, (2) getting rid of price discounts and special offers on the product mentioned in the message, (3) getting rid of low-cost versions of the product mentioned in the message. One was related to reactance, a psychological response to having one's freedom limited.

Results: We found no effect for pricing policy in the message. Frames regarding children and reducing cancer risk moderated some outcomes, showing promise for real-world use. We found differences in support by product and reactance with greatest support and least reactance for tobacco policies, less support and more reactance for alcohol policies, and the least support and most reactance for sugary drinks/foods policies.

Conclusions: Cancer prevention efforts utilizing policy interventions can be informed by the message framing literature. Our results offer insights for cancer prevention advocacy efforts across the UK and USA and highlight that tax versus non-tax approaches to increasing the cost of cancer-causing products result in similar responses from consumers.

Article Summary

Strengths and limitations of this study

- We conducted qualitative formative work and a pilot study to inform the experimental design and stimuli.
- Our team and data represented both the UK and the USA.
- We have strong internal validity given our use of a randomised factorial design.
- Our sample size was based on a power calculation for small effect sizes.
- Testing messages in a controlled experiment likely has limited ecological validity given real-world exposure to multiple messages across multiple channels.

For peer review only - http://bmjopen.bmj.com/site/about/guidelines.xhtml

Funding

This work was supported by Cancer Research UK (C18486/A25644), internal research funds at East Carolina University, UK Medical Research Council (MC_UU_12017/13, MC_UU_12017/15), the Chief Scientist Office of the Scottish Government Health Directorates (SPHSU15), and the U.S. National Institutes of Health (K08AA023290, T32DA043449). The content is solely the responsibility of the authors and does not necessarily represent the official views of the funders.

Competing Interests

The authors report no competing interests.

Data Sharing

Data, codebooks, and interview guides are available in East Carolina University Dataverse, doi:10.15139/S3/IAKUQN, available from: https://dataverse.unc.edu/dataset.xhtml?persistentId=doi:10.15139/S3/IAKUQN

For peer review only

INTRODUCTION

One-third of the risk and burden of cancer is attributed to four common risk factors – use of tobacco, use of alcohol, unhealthy diets, and physical inactivity.[1] These individual behaviours are influenced by a complex array of factors, including people's perceptions and beliefs as well as macro-level drivers such as corporate marketing of unhealthy products including product price.[2 3] Pricing interventions that increase the price of harmful products are an effective measure governments have to help encourage consumers to reduce the consumption of products that contribute to cancer.[4-12] There are multiple ways to increase costs of products. Many alcohol, sugary drinks/foods, and tobacco products can be subject to excise taxes, which levy a specific or proportional fee on a product that is collected as revenue by the levying government. In addition to taxation, emerging research identifies *non-tax* price-raising strategies, including policies that set a minimum product price; mitigation fees that recoup public costs from product use; minimum pack sizes that prevent very cheap product prices; minimum excise taxes that ensure a certain level of taxation; restricting sales to government stores; and bans on product price discounts and other price marketing.[9 13-16] The World Health Organization considers such policies that raise the cost of products "best buys" for preventing and controlling non-communicable diseases.[17]

Despite strong evidence for the effectiveness of price-related policies, the implementation of such policies has been limited, due in part to industry efforts to undermine public and political support.[18-20] Indeed, substantial portions of the public remain sceptical of tax interventions and they are often described as nanny state policies or government overreach.[21-25] Despite this scepticism, support for efforts to protect children and to give everyone a comparable opportunity of achieving a healthy life are broadly supported.[26] This disconnect between a lack

For peer review only - http://bmjopen.bmj.com/site/about/guidelines.xhtml

of public support for price-related policy interventions, yet strong support for protecting people's health represents an opportunity to reframe the debate.

Public health advocates must translate potentially effective policies into ideas that resonate with public concerns.[27] The field of message framing, based in sociology and prospect theory from behavioural economics, suggests that how the information is presented changes how it is received and interpreted.[28 29] There is good evidence showing that the framing of policies in the media or in communication campaigns can play a critical role in the adoption of policies.[25 30-33] For example, in the US, research has suggested that mixing individual and community responsibility frames can reduce counter arguments from more conservatively-oriented members of the public, and linking other popular policies to a proposed policy can be an important strategy (e.g., a tax is connected with financing universal early childhood education or a "public health levy").[26 34 35] Some types of messages can perform well across the left-to-right ideological spectrum including focusing on military readiness, healthcare costs, and reducing obesity-related bullying.[36] However, messages identifying social inequities may perform poorly by invoking discriminatory stereotypes from the more advantaged groups and negative emotions from more disadvantaged groups.[26 37 38] In Scotland, framing minimum unit alcohol pricing policy as a public health and whole-population issue has been crucial to enabling policymakers to adopt policies.[33] Some research also suggests that showing the perceived effectiveness of a policy can have a small, positive effect on policy support.[39]

As such, framing can be an important tool in public health advocacy efforts to gain support for policies that address corporate determinants of health.[40 41] Prior research on framing has enumerated frames used to address health-harming products.[42 43] In addition to understanding the role of the policy and the message's framing, it is important to know if there are differences

For peer review only - http://bmjopen.bmj.com/site/about/guidelines.xhtml

For peer review only

in support by the type of product. Differences in support by the product could inform advocacy campaigns – especially if advocates move from a product-by-product approach to an umbrella approach for cancer-causing products.

To this end, as part of a US National Cancer Institute and Cancer Research UK sponsored Knowledge Integration Sandpit,[44] we formed a bi-national, interdisciplinary team to explore the use of framing in cancer-prevention pricing policies. We hypothesized that (**Hyp_1**) messages about non-tax pricing policies would result in greater levels of support for pricing policies than messages about tax policies given a strong distaste for taxes as a policy intervention;[23] (**Hyp_2**) pricing policies would be differentially supported based on different frames devised through a qualitative development process; and, (**Hyp_3**) tobacco-related policies would be more supported than alcohol- or sugary drinks/foods-related policies.

METHODS

Message frame development and formative interviews

This factorial experimental design contained three stages and a sample drawn from the UK and US public. In stage one, we conducted qualitative interviews with nine adults from the UK and nine adults from the US between February and May 2018. In total, we held 18 interviews, nine in each country (three members of the public, three cancer policy advocates, and three cancer survivors). We leveraged community partnerships and, in the US, student researchers' connections to churches and other organizations. We purposively sampled for diversity by age, gender, and race/ethnicity. Trained interviewers in Scotland and North Carolina conducted interviews in their respective countries using a semi-structured interview guide. Briefly, we asked participants about their initial thoughts, advantages/disadvantages, and

For peer review only - http://bmjopen.bmj.com/site/about/guidelines.xhtml

benefits/harms of four pricing policies (minimum prices, getting rid of coupons or discounts, adding taxes, and mitigation fees). We then asked about level of support for policies targeting specific products: alcohol, sugary drinks/foods, and tobacco. Finally, we asked what arguments for a pricing policy would be persuasive. All interviews were professionally transcribed verbatim, and transcriptions checked by the original interviewer. Transcripts were entered into QSR International's NVivo 12 qualitative data analysis software for thematic analysis.[45] Two researchers read all transcripts in depth and carried out initial coding following the interview guide structure. We then inductively identified recurrent themes for each price policy and compared similarities and differences in views between price policies, products, and countries.

In stage two, we used the results from the formative qualitative interviews and the published literature[22 26 33 43] to develop nine potential frames and iteratively refined them for clarity and readability (a description of the potential frames is available in our repository[46]). For example, based on suggestions from the Robert Wood Johnson Foundation,[26] we developed a frame highlighting individual and community responsibility: "We can all play a role in making healthier communities. Everyone can contribute to this by making healthy choices regarding their own alcohol consumption, but also by supporting increasing taxes on alcohol." We then pilot-tested the frames with 106 UK and US adults in an online survey panel provided by Qualtrics Research Services, 30th October-1st November 2018. Participants reported how well they understood the potential frames on a 3-point scale (1=understood very well, 2=understood fairly well, 3=did not understand well), described each in their own words and ranked the nine frames from most to least compelling. To select the frames for our main experiment, we computed the self-reported mean comprehensibility and the average ranking for each frame, and

For peer review only - http://bmjopen.bmj.com/site/about/guidelines.xhtml

199 double coded (on a 3-point scale) whether participants accurately described the frame. In this
200 step we did not manipulate product or policies; all messages were about alcohol taxes.
201 In the third and final stage, we selected four frames for an experiment. The four frames
202 (Table 1) were adapted to each of three products (alcohol, sugary drinks/foods, tobacco) and
203 each of three policies (taxes, minimum pricing, getting rid of discounting). We created 36
204 messages specific to product, policy, and the four frames about how the policy intervention
205 would (1) reduce strain to healthcare, (2) protect children, (3) prevent cancer, and (4) bring
206 consumption of the product down. All messages are provided in our linked Dataverse repository.

207 **Table 1**. Example frames used in experiment

**Frame Number (short description)**	**Example frame for alcohol product and tax policy**
F_1 (reduce strain on healthcare)	The problems caused by alcohol misuse, such as crime, injuries and addiction, are costly. Increasing taxes on alcohol will reduce the financial strain on our overburdened health care system.
F_2 (protect children)	Alcohol misuse can hurt children's health and wellbeing - both directly (through children drinking alcohol), and indirectly (through adults' misuse of alcohol impacting children). We can protect children by increasing taxes on alcohol.
F_3 (prevent cancer)	Drinking less alcohol can reduce the risk of cancer. Increasing taxes on alcohol is an important way to prevent cancer in our communities.
F_4 (bring consumption down)	Drinking less alcohol can reduce the risk of stroke, high blood pressure, obesity, liver disease, and mental health problems. Increasing taxes on alcohol is an important way to bring drinking levels down.

208
209 **Experimental design and implementation**

210 We conducted an *a-priori* power analysis for the 3x4x3 factorial design using G*Power
211 3.1[47] with power (1 - β) set at 0.80 and α=.05, two-tailed, for a small effect size (Cohen's f
212 =0.10) given prior findings examining similar constructs.[35 36] The power analysis indicated that a
213 sample of 1745 would be needed to detect these small effects. To recruit participants, we
214 engaged Qualtrics Research Services, an online panel service; this approach shows evidence of
215 generalisability for experiments.[48 49] We used quota sampling to include 50% of participants

For peer review only - http://bmjopen.bmj.com/site/about/guidelines.xhtml

from the UK and 50% from the US, equal representation by assigned birth sex, a minimum of 20% of participants with fewer than four years of post-secondary education, and a minimum of 20% of participants reporting having smoked 100 or more cigarettes in one's life. Qualtrics provided each participant "points" in exchange for completing the survey that could be redeemed for incentives such as gift cards. Qualtrics successfully recruited and provided data from 1,850 participants. In this between-subjects experimental factorial design, each participant was randomly assigned to view one of the 36 messages using Qualtrics's advanced block programming. Table 2 shows the experiment design with blue text for the cancer product and red text for the policy.

Table 2. Frame x products x policy factorial design

		**Policy**		
		**T**ax	Getting Rid of **D**iscounting	Min. **P**rice
**Cancer Product**	**A**lcohol	F_1 A T	F_1 A D	F_1 A P
		F_2 A T	F_2 A D	F_2 A P
		F_3 A T	F_3 A D	F_3 A P
		F_4 A T	F_4 A D	F_4 A P
	**S**ugary Drinks/Foods	F_1 S T	F_1 S D	F_1 S P
		F_2 S T	F_2 S D	F_2 S P
		F_3 S T	F_3 S D	F_3 S P
		F_4 S T	F_4 S D	F_4 S P
	**T**obacco	F_1 T T	F_1 T D	F_1 T P
		F_2 T T	F_2 T D	F_2 T P
		F_3 T T	F_3 T D	F_3 T P
		F_4 T T	F_4 T D	F_4 T P

After brief screening and demographic questions, we provided the following prompt: "On the next screen, we will show you a message about a public health policy and a product. After you read the message, we'll ask you questions about what you think about policies about that product. PLEASE READ THE MESSAGE ABOUT THE PRODUCT ON THE NEXT SCREEN. You will not be able to see it again." The participant then viewed one of the 36 messages.

For peer review only

Measures

To assess the impact of the different products, policies, and frames in our messages, we used four dependent variables. Following prior work by Niederdeppe,[35] we first measured policy support with three questions: "How much do you oppose or support the following?" i) Increasing taxes on the product mentioned in the message, ii) Getting rid of price discounts and special offers on the product mentioned in the message, and iii) Getting rid of low-cost versions of the product mentioned in the message. There were five response options ranging from Strongly Oppose (1) to Strongly Support (5) with a neutral point. Each participant answered all three questions regardless of the policy invoked in their message frame. We also measured reactance, a psychological response to having one's freedom limited,[50] to the message using six items adapted from Hall,[51] e.g., "The product mentioned is legal, so the government should stop interfering with it." There were five response options ranging from Strongly Disagree (1) to Strongly Agree (5) with a neutral point, which we averaged into a scale. These items exhibited good internal consistency in our data (McDonald's omega=0.87) and could not be improved by removing any item.[52]

We also measured potential covariates and factors including country (UK, US), and use of alcohol, sugary beverages, and cigarettes in the last 30 days (options: every day, some days, not at all). Full details of measures are available in our repository.

Analysis of Experiment

We assessed whether the: (1) type of policy, (2) product, or (3) frame used in a message was associated support for each type of policy and reactance, as well as interactions among these main predictors. We also assessed interactions between covariates (sex, age, country) and main

For peer review only

For peer review only - http://bmjopen.bmj.com/site/about/guidelines.xhtml

predictors; if they were significant, those covariates were analysed as an additional factor as was the case for country in two of the models. All main effects and interactions were analysed using a 3x4x3 ANOVA. In two of the models, where country was significant, a 2x3x4x3 ANOVA was conducted. When main effects or interactions were significant, we conducted pairwise comparisons using Fisher's least significant differences test. We used a traditional threshold of statistical significance, $p<0.05$ and used two tailed tests. We used SPSS v.25 (IBM, Chicago, IL).

Research undertaken in this project was reviewed by the East Carolina University and Medical Center Institutional Review Board (#17-002904) and University of Glasgow College of Medical, Veterinary & Life Sciences Ethics Committee (#200170074).

Patient and Public Involvement

Patients and the public were not involved in this research.

RESULTS

Formative interviews

We completed formative interviews with eighteen participants, nine in the UK and nine in the US. Interviewees were between ages 24 and 69 years (average age 48), and cancer survivors had a range of cancer experiences including prostate, lung, breast, head and neck, and skin cancers. Interviews lasted between twenty minutes and one hour depending on the interviewees' interest in the subject area. In general, interviewees were more supportive of price policies for tobacco containing commodities than either alcohol or sugary drinks/foods (Table 3).

Table 3: Interviewees' overall support for price policies in general by product type, country, and interviewee type, N=18

Product	UK interviewees									US interviewees									Mean
	Advocates			Survivors			Public			Advocates			Survivors			Public			
Tobacco	9	10	8	8	9	10	1	9	9	8	9	8	8	5	8	7	8	8	7.9
Alcohol	9	10	4	3	7	7	1	5	6	7	6	4	3	5	5	4	8	6	5.6
Sugary Drinks/Foods	10	10	3	8	4	8	3	4	9	5	3	2	7	5	2	5	6	4	5.4

For peer review only - http://bmjopen.bmj.com/site/about/guidelines.xhtml

Note: 1=not supportive, 10=very supportive.

During the interviews it became apparent that participants found it difficult to differentiate between a hypothecated tax and a mitigation fee. The team discussed this issue and decided to drop mitigation fee as a policy in the main experiment. A number of emergent themes were common to all three pricing policies (Table 4). Specifically, participants thought that all of the policies would have an impact on purchasing behaviour with a concomitant health benefit due to reduced consumption, particularly for young people and those on low income. Participants also thought they could have an indirect benefit by drawing attention to the product and the level of potential health harm. For example, a policy advocate and cancer survivor said, respectively:

'*…it would reduce the excessive consumption of these products, it would control the quantities in which these products are consumed, and it would mean the consumer… is more aware of their purchasing of the products.*' (Policy advocate)

'The biggest benefit is that it will make the person aware – some people drink and eat things they're not aware of, they don't look into it – and if they see that there's a higher tax, and they start recognising that, they're going to find out why and it might change their behaviour.' (Cancer survivor)

[Table 4 appears on next page.]

For peer review only - http://bmjopen.bmj.com/site/about/guidelines.xhtml

Table 4. Themes identified in qualitative interviews

Common for all 3 pricing policies	Specific to minimum price	Specific to getting rid of discounts	Specific to tax
**Supportive arguments/ advantages**			
• Health benefit<br>• Greatest benefit for young<br>• Greatest benefit for low SES<br>• Will affect purchasing behaviour<br>• Will highlight real cost of consumption/health harms<br>• More supportive where there is clear evidence of cancer links	• A deterrent or nudge<br>• Consistency and fairness<br>• Targets most harmful consumption<br>• There is evidence to support it	• Price promotions most strongly influence purchasing<br>• Promotions encourage over consumption<br>• Will protect industry and retailer profits	• Could prompt reformulation<br>• Could be a form of health insurance<br>• Source of public revenue to fund social policy programmes/ health services<br>• An effective way of moderating behaviour
**Unsupportive arguments/ disadvantages**			
• Effectiveness will depend on individual response<br>• Needs to be prohibitively high to change consumption<br>• Unfair on responsible consumers<br>• Addicts will pay more<br>• Could create a black market (US specific)<br>• Regressive<br>• Bad for retailers/ producers<br>• Simplistic – not enough	• Contravenes EU law<br>• Public do not understand/ will not support it<br>• Revenue raised goes to industry<br>• Too specific for food<br>• Will not impact population level drinking	• Lost opportunity to apply to healthy foods<br>• Will push prices up across the board<br>• Inappropriate to interfere in pricing	• Nanny statist - interference in freedom of choice<br>• Political cost – unpopular<br>• Reformulation could prompt increased consumption<br>• Negative unintended consequences - substitutes may be equally unhealthy<br>• Will not work if funds are not ring-fenced

For peer review only - http://bmjopen.bmj.com/site/about/guidelines.xhtml

Some key disadvantages were also highlighted across all policies: (1) that their effectiveness would depend on the scale of the price increase and how individuals responded; (2) that they are potentially regressive; and, (3) that they are unfair to responsible consumers and to those who are dependent on these substances. Participants were especially concerned about the impact on individuals dependent on these products with limited resources, particularly in relation to alcohol consumption. Yet, this concern also applied to tobacco and sugary drinks/foods, which were both deemed to have addictive qualities. Quotes illustrating these disadvantages include:

> *'The effect on health does depend on what decisions people make in response to it. And whether it follows what we expect. And does it reduce purchase of those products, does it push people to other products or not?'* (Policy advocate)

> *'I think they [people with limited incomes] would then be more likely to stop buying food, to stop heating their house, or to stop paying their rent or, you know, whatever, in order to prioritize the alcohol consumption.'* (General public)

There were a number of unique themes arising for each price policy (Table 3). For example, although discounts and price promotions were thought to be the strongest influencer of purchasing behaviour and to encourage over consumption, interviewees thought they should be refocused on healthier foods and drinks rather than replaced. For minimum unit price policies specifically, interviewees noted that an advantage of such policies is that they target the most harmful patterns of consumption, i.e., the highest levels of consumption which can result in anti-social behaviour. Taxation alone prompted interviewees to talk about nanny state arguments and inappropriate government intervention in the market. This response was more marked in the UK

For peer review only

For peer review only - http://bmjopen.bmj.com/site/about/guidelines.xhtml

than the US and might be explained by the fact that the timing of the interviews coincided with the implementation of the UK Soft Drinks Industry Levy ("Sugar Tax") in April 2018.

The frames that interviewees thought would be most persuasive and result in increased support for price policies could be summarised as those that: (1) draw a very clear link to potential health harm from consuming the product; (2) focus on a positive health impact arising from the policy, particularly protection of children; (3) emphasise the impact on health services in terms of reduced costs and the potential to generate revenue for reinvestment; and, (4) make the policies meaningful in terms of the anticipated benefit for individuals, in straight forward language. Less convincing frames were those that: (1) employ threats or scare tactics; (2) seem condescending or nanny statist; (3) use jargon or statistics; and, (4) set unrealistic expectations of behaviour change for individuals. We used these findings to inform the experimental stimuli, results for which are presented next.

Main experimental effects

Participants were 1,850 adults (ages 18-86, M_{age}=38.56, SD=13.56). The sample was evenly split across the countries of interest (50.1% from the UK) and sex assigned at birth (50.2% female). Within the sample, 34.7% graduated from college or university, 26.4% attended some college or university, 28.9% had graduated or completed secondary or high school, and the remainder (7.6%) attended some high school or less. Table 4 shows the pattern of results.

[Table 5 appears on the next page.]

For peer review only - http://bmjopen.bmj.com/site/about/guidelines.xhtml

Table 5. Summary of significance direction of main effects and interactions between factors and dependent variables, 2019, N=1,850, UK and US adults

**Factors**	**Dependent variables**			
	**Support for raising taxes**	**Support for getting rid of price discounts and special offers**	**Support for getting rid of low-cost version of product**	**Psychological reactance**
Policy (taxes, minimum pricing, getting rid of discounting)	• Main: NS<br>• Interactions: NS	• Main: NS<br>• Interactions: NS	• Main: NS<br>• Interactions: NS	• Main: NS<br>• Interactions: NS
Frame (1. reduce strain to healthcare, 2. protect children, 3. prevent cancer, and 4. bring consumption of the product down)	• Main: NS<br>• Interactions: NS	• Main: Higher support with Frame 2 (Children) and 3 (Prevent Cancer) than 4 (Reduce Use)<br>• Interaction: NS	• Main: NS<br>• Interaction: For participants receiving Frame 3 (Prevent Cancer) support was higher for minimum pricing policy in message than for tax policy in message<br>• For participants receiving Frame 4 (Reduce Use) differences in support by policy in message	• Main: NS<br>• Interaction: Frame 2 (Children) lowered reactance for UK participants more than US participants
Product (alcohol, sugary drink/food, tobacco)	• Main: Tobacco: Highest; Alcohol: Lower; Sugar: Lowest;<br>• Interaction: Tobacco higher for UK than US	• Main: Tobacco: Highest; Alcohol: Lower; Sugar: Lowest<br>• Interaction: NS	• Main: Tobacco: Highest; Alcohol: Lower; Sugar: Lowest<br>• Interaction: NS	• Main: Sugar: Highest; Alcohol: Lower; Tobacco: Lowest<br>• Interaction: Sugar and Tobacco lower for UK than US
Country (UK, USA)†	• Main: UK: Higher; US: Lower<br>• Interaction: NS	• Main: NS<br>• Interaction: NS	• Main: NS<br>• Interaction: NS	• Main: US: Higher; UK: Lower<br>• Interaction: NS

For peer review only - http://bmjopen.bmj.com/site/about/guidelines.xhtml

Note: Sugar = Sugary Drinks/Foods, NS=not statistically significant; † Covariate assessed as a factor given significant interactions with other factors; interactions assessed were between covariates (sex, age, country) and main predictors (policy, frame, product; if they were significant, those covariates were analysed as an additional factor as was the case for country in two of the models

Dependent variable 1: Support for increasing taxes on the product mentioned in the message

For the first outcome, there was not a significant main effect for policy (i.e., taxes, minimum pricing, getting rid of discounting) or frame (i.e., reduce strain to healthcare, protect children, prevent cancer, and bring consumption of the product down). There was a significant main effect for product (F[2, 1767]=45.71, p<0.001) and country (F[1, 1767]=26.63, p<0.001). Pairwise comparisons for product revealed significant differences across all three products (M_{sugar}=2.76, SD=1.26; $M_{alcohol}$=3.07, SD=1.30; $M_{tobacco}$=3.47; SD=1.37); participants receiving a message regarding sugary drinks/foods had the lowest and participants receiving a message regarding tobacco endorsed the highest levels of support for increasing taxes on the product. Pairwise comparisons for country revealed that participants from the UK (M_{UK}=3.257, SD=1.33) endorsed higher levels of support for increasing taxes compared to participants from the US (M_{US}=2.937, SD=1.34). There was also a significant two-way interaction: product x country, F(2, 1767)=5.67, p<0.01. Specifically, among participants receiving a sugary drinks/foods product message, those from the UK endorsed higher levels of support for increasing taxes on the product (M_{UK}=3.08, SD=1.28) compared to US participants (M_{US}=2.51, SD=1.19). Similarly, among participants receiving a tobacco product message, those from the UK endorsed higher levels of support for increasing taxes on the product (M_{UK}=3.69, SD=1.32) compared to US participants (M_{US}=3.24, SD=1.38). Three-way interactions were not significant.

Dependent variable 2: Support for getting rid of price discounts and special offers on the product mentioned in the message

For peer review only - http://bmjopen.bmj.com/site/about/guidelines.xhtml

For the second outcome, there was not a significant main effect for policy (i.e., taxes, minimum pricing, getting rid of discounting). There was a significant main effect for frame (F[3, 1803]=2.62, p<0.05) and message product (F[2, 1803]=53.80, p <0.001). Participants receiving frames 2 (protect children, M_{frame2}=3.25, SD=1.37) or 3 (prevent cancer, M_{frame3}=3.24, SD=1.35) endorsed higher levels of support for getting rid of price discounts and special offers than participants who received frame 4 (bring consumption down, M_{frame4}=3.04, SD=1.36). Pairwise comparisons for product revealed significant differences across all three products ($M_{sugar=}$2.85, SD=1.28; $M_{alcohol}$=3.05, SD=1.34; $M_{tobacco}$=3.59; SD=1.35); participants receiving a message regarding sugary drinks/foods and participants receiving a message regarding tobacco endorsed the lowest and highest levels of support for getting rid of price discounts and special offers on the product, respectively. There were no significant two-way or three-way interactions.

Dependent variable 3: Support for getting rid of low-cost versions of the product mentioned in the message

For the third outcome, there was not a significant main effect for policy (i.e., taxes, minimum pricing, getting rid of discounting) or frame (i.e., reduce strain to healthcare, protect children, prevent cancer, and bring consumption of the product down). There was a significant main effect for message product (F(2, 1802)=60.34, p<0.001). Pairwise comparisons revealed significant differences across all three products ($M_{sugar=}$2.82, SD=1.26; $M_{alcohol}$=3.08, SD=1.29; $M_{tobacco}$=3.60; SD=1.34), such that participants receiving a message regarding sugary drinks/foods and participants receiving a message regarding tobacco endorsed the lowest and highest levels of support for getting rid of low-cost versions of the product, respectively. There was also a significant two-way interaction: frame x policy, F(6, 1802)=2.24, p<0.05. Specifically, among participants receiving frame 3 (prevent cancer), participants who received

For peer review only - http://bmjopen.bmj.com/site/about/guidelines.xhtml

the policy related to minimum pricing endorsed higher levels of support for getting rid of low-cost versions of the product ($M_{minprice}$=3.36, *SD*=1.28) compared to those receiving the policy related to increasing taxes (M_{tax}=3.05, *SD*=1.38). Additionally, among participants receiving frame 4 (bring consumption down), there were significant differences in support for getting rid of low-cost products between participants who received policies related to getting rid of discounting ($M_{nodiscounting}$=3.23, *SD*=1.30) and minimum pricing ($M_{minprice}$=3.16, *SD*=1.37), and participants who received the policy related to increasing taxes (M_{tax}=2.76, *SD*=1.29). There were no significant three-way interactions.

Dependent variable 4: Reactance

For the last outcome, there was not a significant main effect for policy or frame on reactance (i.e., the extent to which one perceives their freedoms to be limited). There was a significant main effect for message product (F[2, 1769]=30.74, p<0.001) and country (F[1, 1769]=7.21, p<0.01). Pairwise comparisons for product revealed significant differences across all three products ($M_{tobacco}$=2.72; *SD*=0.99; $M_{alcohol}$=2.92, *SD*=0.95; $M_{sugar=}$3.14, *SD*=0.93), such that participants receiving a message regarding tobacco and participants receiving a message regarding sugary drinks/foods, indicated the lowest and highest levels of reactance, respectively. Pairwise comparisons for country revealed that participants from the US (M_{US}=2.99, *SD*=0.97) endorsed higher levels of reactance compared to participants from the UK (M_{UK}=2.87, *SD*=0.97). There were two significant two-way interactions: product x country, F(2, 1769)=4.09, p<0.05 and frame x country F(3, 1769)=2.84, p<0.05. Specifically, among those that received a message regarding tobacco, participants from the US endorsed significantly higher levels of reactance (M_{US}=2.838, *SD*=0.98) compared to participants from the UK (M_{UK}=2.606, *SD*=0.98). Similarly, among those receiving a message regarding sugary drinks/foods, participants from the US

For peer review only

For peer review only - http://bmjopen.bmj.com/site/about/guidelines.xhtml

endorsed significantly higher levels of reactance (M_{US}=3.24, SD=0.91) compared to participants from the UK (M_{UK}=3.05, SD=0.93). Among those who received frame 2 (protect children), participants from the UK endorsed lower levels of reactance (M_{UK}=2.71, SD=0.97) than those from the US (M_{US}=3.01, SD=0.93).

Lastly, there was also a significant three-way interaction: product x policy x country, (F[4, 1769]=2.97, p<0.05). Among those receiving a message regarding alcohol, those from the UK endorsed significantly higher levels of reactance (M_{UK}=3.05, SD=0.92) compared to participants from the US (M_{US}=2.78, SD=0.94) receiving the getting rid of discounting policy. In contrast, among those receiving a message regarding sugary drinks/foods, those from the US endorsed significantly higher levels of reactance (M_{US}=3.39, SD=0.92) compared to participants from the UK (M_{UK}=3.07, SD=0.95) for those receiving the policy related to increasing taxes. Among those receiving a message regarding tobacco, those from the US endorsed significantly higher levels of reactance (M_{US}=2.93, SD=1.05) compared to participants from the UK (M_{UK}=2.48, SD=0.95) receiving the getting rid of discounting policy.

DISCUSSION

Principal findings

This research aimed to explore whether the specific content of a message about cancer-prevention price policies was associated with higher public support and lower reactance for those policies. We had three main hypotheses. First, we hypothesized that messages about non-tax pricing policies would result in more support than messages about tax policies. Our results did not provide evidence for this hypothesis: Not one of the three policies tested (taxes, minimum pricing, and getting rid of discounts) showed a statistically significant impact on support in our experiment. Regarding our second hypothesis, that support for a particular pricing policy would

change based on the framing of the message, we found some evidence. Specifically, of the frames we tested, which were selected based on their performance in pilot testing, our findings indicate that frames relating to children show promise. We found evidence to support our third hypothesis that messages about tobacco products would result in more support for raising the price of products than messages about alcohol or sugary drinks/foods products. Product in the message also changed reactance, which was highest for sugary drinks/foods and lowest for tobacco. Finally, we found statistically significant differences in responses to messages by country (e.g., support for alcohol pricing interventions was lower in the US than in the UK).

Strengths and weaknesses

The strong internal validity from the experimental design, qualitative formative work, and international scope of this research must be balanced against limitations. First, this study used an online convenience sample from a panel provider service, Qualtrics, which may limit its external validity given the participants are not representative of their respective countries, particularly in tobacco use and educational attainment. However, prior research indicates that convenience samples like ours generalize well when used in experimental studies rather than studies of prevalence.[48] Second, we assessed the impact of a single exposure of a message displayed on a screen. This does not reflect the real-world environment where messages about policies may be delivered from multiple sources, repeated, and seen on multiple communication channels. Additionally, a limitation to survey research is that participants can interpret response scales differently which can impact data and interpretations. Thus, participants were provided with a wider range of responses (e.g., 5-point Likert scales) and category labels to minimize these variations. Third, further work should explore equity implications of our messaging

For peer review only - http://bmjopen.bmj.com/site/about/guidelines.xhtml

approaches – especially given growing evidence regarding the pro-equity effect of pricing interventions.[53]

Results in context

The process of policy development as well as policy making decisions are ultimately taken by politicians on behalf of their constituents (the public); in this context, the scientific evidence- and value-bases play important roles.[54] While the evidence-base for different types and targets of price policies on behaviour is rapidly expanding,[55-60] the difficulty of increasing public support and consequently political support for pricing interventions has received less attention. The relatively low levels of public support have previously been highlighted.[22 61] Our findings provide further evidence to suggest limited support among the public for pricing interventions overall, given relatively low endorsements of support on our support dependent variables. However, given the mean scores, the average participant was also not that opposed. Furthermore, a policy message that specifically referenced a particular type of price policy did not result in more support for that type of price policy compared to other types.

Prior research has examined the role of framing in public health efforts, and our findings demonstrate ways to best utilize media advocacy approaches[62] by minimizing reactance from the public and promoting support of evidence-based policies. Specifically, our frames were all selected based on the prior literature and their performance in formative work. Particular value may come from framing messages around the protection of children. This is consistent with previous work.[26] Previous work has also shown that the product under consideration influences policy support.[63] Our experimental findings offer further evidence of this, showing that participants rated support of pricing interventions highest on messages for tobacco products, followed by alcohol, and lastly by sugary drinks/foods. Finally, our findings highlight some

For peer review only - http://bmjopen.bmj.com/site/about/guidelines.xhtml

notable differences by country, which may reflect more general differences in acceptance of policies, healthcare systems, and political developments at the time of our study (e.g., 'Brexit').

Implications for Public Health Policy and Practice

Our findings have several implications for messaging about pricing policies and for policy research, particularly with the aim of garnering support (across stakeholder groups from public, civil society, media, and politicians) to develop and implement price policies. First, the evidence of differences by product may indicate that efforts to "denormalize" tobacco products may have yet to translate to alcohol and sugary drinks/foods efforts. Policy advocates should consider if lessons learned from tobacco control can and should be applied to alcohol and sugary drinks/foods. It is worth highlighting that denormalization can present the risk of increasing stigma, which has been a criticism in the area of tobacco.[64] Second, our findings suggest the importance of bridging the research-practice gap between the literature on framing and advocacy organizations' messaging. Frames that include a focus on protecting children seem to show particular promise; however, our four frames showed few significant difference in their overall performance. This is likely because we used a robust pilot testing process to develop the frames, and we only selected the best performing frames for use in the main experiment. Practitioners should consider use of the four frames tested here. Third, practitioners should consider lessons learned from other countries, which may help with identifying particular challenges and successes regarding cancer prevention.

Conclusions

In our online experiment with participants from the UK and US, we found no significant differences in price policy message support or reactance based on the type of proposed policy, suggesting advocates can recommend policies that raise prices through both tax and non-tax

For peer review only - http://bmjopen.bmj.com/site/about/guidelines.xhtml

means. Results also indicate that framing messages focused on protecting children had increased support and lowered reactance in some combinations of experimental stimuli. Public health policy advocates should consider using frames like those we tested in ongoing efforts to develop, adopt, and implement price policies that reduce use of cancer-causing products. This study also indicates that lessons learned from efforts to build public support for addressing tobacco use may be needed to successfully address alcohol and sugary drinks/products, since support for pricing policies for these products were generally lower than for tobacco price policies. Public health practitioners and advocates should consider using tested messages when working to advance pricing policies that reduce consumption of harmful products marketed by powerful corporations.

Author Contributions

JGLL, RNC, EMT, DIC conceived the study. JGLL, CHB, RNC, EMT, TI, SH, SDG, and DIC designed the study. JVC (quantitative) and CHB (qualitative) conducted the data analysis with input from EMT and SH, respectively. PMS conducted analyses of the pilot study with input from RNC. JGLL, CHB, RNC, EMT, PMS, SDG, and DIC developed the stimuli. JGLL led the manuscipt drafting; all authors provided critical input on the interpretation of the results, writing of the manuscript, and approved the final version.

Human Participant Protections

Research undertaken in this project was reviewed by the East Carolina University and Medical Center Institutional Review Board (#17-002904) and University of Glasgow College of Medical, Veterinary & Life Sciences Ethics Administration Committee (#200170074).

Acknowledgements

We thank Cancer Research UK and the US National Cancer Institute Health Behaviours Research Branch for the opportunity to participate in their jointly hosted 2017 “sandpit” workshop. We thank Israel M. Mendez for his help with the data collection and project management in the US. An earlier version of this paper was presented in a dissemination event held at the Royal Institution in London in November 2019.

For peer review only

REFERENCES

1. Parkin DM, Boyd L, Walker LC. 16. The fraction of cancer attributable to lifestyle and environmental factors in the UK in 2010. *Br J Cancer* 2011;105 Suppl 2:S77-81. doi: 10.1038/bjc.2011.489 [published Online First: 2011/12/14]

2. Madureira Lima J, Galea S. Corporate practices and health: a framework and mechanisms. *Global Health* 2018;14(1):21. doi: 10.1186/s12992-018-0336-y [published Online First: 2018/02/17]

3. McKee M, Stuckler D. Responding to the Corporate and Commercial Determinants of Health. *Am J Public Health* 2018:e1-e4. doi: 10.2105/ajph.2018.304510 [published Online First: 2018/07/20]

4. Contreary KA, Chattopadhyay SK, Hopkins DP, et al. Economic Impact of Tobacco Price Increases Through Taxation: A Community Guide Systematic Review. *Am J Prev Med* 2015;49(5):800-08. doi: 10.1016/j.amepre.2015.04.026

5. IARC. Effectiveness of tax and price policies for tobacco control. IARC handbooks of cancer prevention. Lyon, France: International Agency for Research on Cancer, 2011.

6. Wagenaar AC, Salois MJ, Komro KA. Effects of beverage alcohol price and tax levels on drinking: a meta-analysis of 1003 estimates from 112 studies. *Addiction* 2009;104(2):179-90. doi: 10.1111/j.1360-0443.2008.02438.x [published Online First: 2009/01/20]

7. Powell LM, Chriqui JF, Khan T, et al. Assessing the potential effectiveness of food and beverage taxes and subsidies for improving public health: a systematic review of prices, demand and body weight outcomes. *Obesity reviews : an official journal of the*

For peer review only - http://bmjopen.bmj.com/site/about/guidelines.xhtml

International Association for the Study of Obesity* 2013;14(2):110-28. doi: 10.1111/obr.12002 [published Online First: 2012/11/24]

8. Burton R, Henn C, Lavoie D, et al. A rapid evidence review of the effectiveness and cost-effectiveness of alcohol control policies: an English perspective. *Lancet* 2017;389(10078):1558-80. doi: 10.1016/s0140-6736(16)32420-5 [published Online First: 2016/12/07]

9. Boniface S, Scannell JW, Marlow S. Evidence for the effectiveness of minimum pricing of alcohol: a systematic review and assessment using the Bradford Hill criteria for causality. *BMJ Open* 2017;7(5):e013497. doi: 10.1136/bmjopen-2016-013497 [published Online First: 2017/06/08]

10. Eyles H, Ni Mhurchu C, Nghiem N, et al. Food pricing strategies, population diets, and non-communicable disease: a systematic review of simulation studies. *PLoS Med* 2012;9(12):e1001353. doi: 10.1371/journal.pmed.1001353 [published Online First: 2012/12/15]

11. Afshin A, Penalvo JL, Del Gobbo L, et al. The prospective impact of food pricing on improving dietary consumption: A systematic review and meta-analysis. *PLoS One* 2017;12(3):e0172277. doi: 10.1371/journal.pone.0172277 [published Online First: 2017/03/02]

12. Teng AM, Jones AC, Mizdrak A, et al. Impact of sugar-sweetened beverage taxes on purchases and dietary intake: Systematic review and meta-analysis. *Obesity reviews : an official journal of the International Association for the Study of Obesity* 2019;20(9):1187-204. doi: 10.1111/obr.12868 [published Online First: 2019/06/21]

For peer review only

For peer review only - http://bmjopen.bmj.com/site/about/guidelines.xhtml

586 13. Golden SD, Farrelly MC, Luke DA, et al. Comparing projected impacts of cigarette floor price and excise tax policies on socioeconomic disparities in smoking. *Tob Control* 2016;25(Suppl 1):i60-i66. doi: 10.1136/tobaccocontrol-2016-053230 [published Online First: 2016/10/05]

590 14. Golden SD, Smith MH, Feighery EC, et al. Beyond excise taxes: a systematic review of literature on non-tax policy approaches to raising tobacco product prices. *Tob Control* 2016;25(4):377-85. doi: 10.1136/tobaccocontrol-2015-052294 [published Online First: 2015/09/24]

594 15. Hiscock R, Augustin NH, Branston JR, et al. Standardised packaging, minimum excise tax, and RYO focussed tax rise implications for UK tobacco pricing. *PLoS One* 2020;15(2):e0228069. doi: 10.1371/journal.pone.0228069 [published Online First: 2020/02/14]

598 16. Kerr WC, Williams E, Greenfield TK. Analysis of Price Changes in Washington Following the 2012 Liquor Privatization. *Alcohol Alcohol* 2015;50(6):654-60. doi: 10.1093/alcalc/agv067 [published Online First: 2015/06/26]

601 17. World Health Organization. Tackling NCDs: 'Best buys' and other recommended interventions for the prevention and control of noncommunicable diseases Geneva: World Health Organization; 2017 [Available from: https://www.who.int/ncds/management/best-buys/en/ accessed January 21 2020.

605 18. Berg CJ, Solomon M, Barkley A, et al. Tobacco Taxes in the Southeastern US States: Views from Former Legislators. *Health behavior and policy review* 2015;2(5):333-42. doi: 10.14485/hbpr.2.5.1 [published Online First: 2015/08/04]

For peer review only - http://bmjopen.bmj.com/site/about/guidelines.xhtml

19. Katikireddi SV, Hilton S. How did policy actors use mass media to influence the Scottish alcohol minimum unit pricing debate? Comparative analysis of newspapers, evidence submissions and interviews. *Drugs (Abingdon, England)* 2015;22(2):125-34. doi: 10.3109/09687637.2014.977228 [published Online First: 2015/06/06]

20. Holmes CB, King BA, Babb SD. Stuck in Neutral: Stalled Progress in Statewide Comprehensive Smoke-Free Laws and Cigarette Excise Taxes, United States, 2000-2014. *Prev Chronic Dis* 2016;13:E80. doi: 10.5888/pcd13.150409 [published Online First: 2016/06/17]

21. Stockwell T. Minimum unit pricing for alcohol: the most cost-effective of cancer prevention strategies? *Expert review of anticancer therapy* 2017;17(11):981-83. doi: 10.1080/14737140.2017.1381565 [published Online First: 2017/09/21]

22. Barry CL, Niederdeppe J, Gollust SE. Taxes on sugar-sweetened beverages: results from a 2011 national public opinion survey. *Am J Prev Med* 2013;44(2):158-63. doi: 10.1016/j.amepre.2012.09.065 [published Online First: 2013/01/22]

23. Petrescu DC, Hollands GJ, Couturier DL, et al. Public Acceptability in the UK and USA of Nudging to Reduce Obesity: The Example of Reducing Sugar-Sweetened Beverages Consumption. *PLoS One* 2016;11(6):e0155995. doi: 10.1371/journal.pone.0155995 [published Online First: 2016/06/09]

24. Eykelenboom M, van Stralen MM, Olthof MR, et al. Political and public acceptability of a sugar-sweetened beverages tax: a mixed-method systematic review and meta-analysis. *Int J Behav Nutr Phys Act* 2019;16(1):78. doi: 10.1186/s12966-019-0843-0 [published Online First: 2019/09/06]

For peer review only

25. Weishaar H, Dorfman L, Freudenberg N, et al. Why media representations of corporations matter for public health policy: a scoping review. *BMC Public Health* 2016;16:899. doi: 10.1186/s12889-016-3594-8 [published Online First: 2016/09/01]

26. RWJF. A new way to talk about the social determinants of health 2010 [Available from: http://www.rwjf.org/content/dam/farm/reports/reports/2010/rwjf63023 accessed April 20 2015.

27. Smith K. Beyond evidence based policy in public health: the interplay of ideas. New York, NY: Palgrave 2013.

28. Goffman E. Frame analysis: An essay on the organization of experience. Cambridge, MA: Harvard University Press 1974.

29. Tversky A, Kahneman D. The framing of decisions and the psychology of choice. *Science* 1981;211(4481):453-8. [published Online First: 1981/01/30]

30. Benford RD, Snow DA. Framing processes and social movements: An overview and assessment. *Annual review of sociology* 2000;26(1):611-39.

31. Cacciatore MA, Scheufele DA, Iyengar S. The end of framing as we know it… and the future of media effects. *Mass Communication and Society* 2016;19(1):7-23.

32. Koon AD, Hawkins B, Mayhew SH. Framing and the health policy process: a scoping review. *Health Policy Plan* 2016;31(6):801-16.

33. Katikireddi SV, Bond L, Hilton S. Changing policy framing as a deliberate strategy for public health advocacy: a qualitative policy case study of minimum unit pricing of alcohol. *Milbank Q* 2014;92(2):250-83. doi: 10.1111/1468-0009.12057 [published Online First: 2014/06/04]

For peer review only - http://bmjopen.bmj.com/site/about/guidelines.xhtml

34. Purtle J, Langellier B, Le-Scherban F. A case study of the Philadelphia sugar-sweetened beverage tax policymaking process: Implications for policy development and advocacy. *J Public Health Manag Pract* 2018;24(1):4-8. doi: 10.1097/phh.0000000000000563 [published Online First: 2017/03/04]

35. Niederdeppe J, Roh S, Shapiro MA. Acknowledging individual responsibility while emphasizing social determinants in narratives to promote obesity-reducing public policy: a randomized experiment. *PLoS One* 2015;10(2):e0117565. doi: 10.1371/journal.pone.0117565 [published Online First: 2015/02/24]

36. Gollust SE, Niederdeppe J, Barry CL. Framing the consequences of childhood obesity to increase public support for obesity prevention policy. *Am J Public Health* 2013;103(11):e96-102. doi: 10.2105/ajph.2013.301271 [published Online First: 2013/09/14]

37. Landrine H, Corral I. Targeting Cancer Information to African Americans: The Trouble With Talking About Disparities. *J Health Commun* 2014:1-8. doi: 10.1080/10810730.2014.920061 [published Online First: 2014/11/21]

38. Friedman AL, Uhrig J, Poehlman J, et al. Promoting sexual health equity in the United States: implications from exploratory research with African-American adults. *Health Educ Res* 2014;29(6):993-1004. doi: 10.1093/her/cyu003 [published Online First: 2014/03/04]

39. Reynolds JP, Pilling M, Marteau TM. Communicating quantitative evidence of policy effectiveness and support for the policy: Three experimental studies. *Soc Sci Med* 2018;218:1-12. doi: 10.1016/j.socscimed.2018.09.037 [published Online First: 2018/10/13]

For peer review only - http://bmjopen.bmj.com/site/about/guidelines.xhtml

40. Jacoby WG. Issue framing and public opinion on government spending. *Am J Pol Sci* 2000:750-67.

41. Wallack L, Dorfman L. Media advocacy: a strategy for advancing policy and promoting health. *Health Educ Q* 1996;23(3):293-317. [published Online First: 1996/08/01]

42. Roth AL, Dunsby J, Bero LA. Framing processes in public commentary on US federal tobacco control regulation. *Social Studies of Science* 2003;33(1):7-44.

43. Niederdeppe J, Gollust SE, Jarlenski MP, et al. News coverage of sugar-sweetened beverage taxes: pro- and antitax arguments in public discourse. *Am J Public Health* 2013;103(6):e92-8. doi: 10.2105/ajph.2012.301023 [published Online First: 2013/04/20]

44. National Cancer Institute Behavioral Research Program. NCI-CRUK Sandpit Workshops 2017 [Available from: https://cancercontrol.cancer.gov/brp/hbrb/sandpit.html accessed December 23 2019.

45. Braun V, Clarke V. Thematic analysis. In: Cooper H, Camic PM, Long DL, et al., eds. APA handbook of research methods in psychology: Research designs: Quantitative, qualitative, neuropsychological, and biological. Washington, DC: American Psychological Association 2012.

46. Lee JG, Conway DI, Carey RN, et al. Dataverse file: Candidate frames tested in pilot project 2020 [Available from: https://dataverse.unc.edu/file.xhtml?fileId=7513206 accessed October 29 2020.

47. Faul F, Erdfelder E, Lang AG, et al. G*Power 3: a flexible statistical power analysis program for the social, behavioral, and biomedical sciences. *Behav Res Methods* 2007;39(2):175-91. [published Online First: 2007/08/19]

For peer review only

697 48. Jeong M, Zhang D, Morgan JC, et al. Similarities and Differences in Tobacco Control Research Findings From Convenience and Probability Samples. *Ann Behav Med* 2019;53(5):476-85. doi: 10.1093/abm/kay059 [published Online First: 2018/07/28]

700 49. Brandon DM, Long JH, Loraas TM, et al. Online instrument delivery and participant recruitment services: Emerging opportunities for behavioral accounting research. *Behavioral Research in Accounting* 2013;26(1):1-23.

703 50. Brehm SS, Brehm JW. Psychological reactance: A theory of freedom and control: Academic Press 1981.

705 51. Hall MG, Sheeran P, Noar SM, et al. Reactance to Health Warnings Scale: Development and Validation. *Ann Behav Med* 2016;50(5):736-50. doi: 10.1007/s12160-016-9799-3 [published Online First: 2016/06/24]

708 52. Hayes AF. My Macros and Code for SPSS and SAS: OMEGA 2019 [Available from: http://afhayes.com/spss-sas-and-mplus-macros-and-code.html accessed December 23 2019.

711 53. Smith CE, Hill SE, Amos A. Impact of population tobacco control interventions on socioeconomic inequalities in smoking: a systematic review and appraisal of future research directions. *Tob Control* 2020 doi: 10.1136/tobaccocontrol-2020-055874 [published Online First: 2020/10/01]

715 54. Oliver K, Cairney P. The dos and don'ts of influencing policy: a systematic review of advice to academics. *Palgrave Communications* 2019;5(1):21. doi: 10.1057/s41599-019-0232-y

717 55. Wagenaar AC, Tobler AL, Komro KA. Effects of alcohol tax and price policies on morbidity and mortality: a systematic review. *Am J Public Health* 2010;100(11):2270-8. doi: 10.2105/ajph.2009.186007 [published Online First: 2010/09/25]

For peer review only - http://bmjopen.bmj.com/site/about/guidelines.xhtml

56. Bennett R, Zorbas C, Huse O, et al. Prevalence of healthy and unhealthy food and beverage price promotions and their potential influence on shopper purchasing behaviour: A systematic review of the literature. *Obesity reviews : an official journal of the International Association for the Study of Obesity* 2020;21(1):e12948. doi: 10.1111/obr.12948 [published Online First: 2019/10/22]

57. Pimpin L, Sassi F, Corbould E, et al. Fiscal and pricing policies to improve public health: a review of the evidence. London, UK: Public Health England, 2018.

58. Martin L, Bauld L, Angus K. Rapid evidence review: The impact of promotions on high fat, sugar and salt (HFSS) food and drink on consumer purchasing and consumption behaviour and the effectiveness of retail environment interventions. Edinburgh: NHS Health Scotland, 2017.

59. Whitehead R, Brown L, Riches E, et al. Rapid evidence review: Strengths and limitations of tobacco taxation and pricing strategies. Edinburgh: NHS Health Scotland, 2018.

60. World Health Organization Regional Office for Europe. Using price policies to promote healthier diets. Copenhagen: WHO Regional Office for Europe 2015.

61. Dorfman L. Talking about sugar sweetened-beverage taxes: will actions speak louder than words? *Am J Prev Med* 2013;44(2):194-5. doi: 10.1016/j.amepre.2012.11.002 [published Online First: 2013/01/22]

62. Dorfman L, Krasnow ID. Public health and media advocacy. *Annu Rev Public Health* 2014;35:293-306. doi: 10.1146/annurev-publhealth-032013-182503 [published Online First: 2013/12/18]

63. Diepeveen S, Ling T, Suhrcke M, et al. Public acceptability of government intervention to change health-related behaviours: a systematic review and narrative synthesis. *BMC*

For peer review only

For peer review only - http://bmjopen.bmj.com/site/about/guidelines.xhtml

Public Health 2013;13:756. doi: 10.1186/1471-2458-13-756 [published Online First: 2013/08/21]

64. Antin TMJ, Annechino R, Hunt G, et al. The Gendered Experience of Smoking Stigma: Implications for Tobacco Control. *Crit Public Health* 2017;27(4):443-54. doi: 10.1080/09581596.2016.1249825 [published Online First: 2017/01/01]

For peer review only

For peer review only - http://bmjopen.bmj.com/site/about/guidelines.xhtml

STROBE 2007 (v4) Statement—Checklist of items that should be included in reports of *cross-sectional studies*

As there is no applicable checklist for a factorial design, we are utilizing the STROBE statement.

Section/Topic	Item #	Recommendation	Reported on page #
**Title and abstract**	1	(*a*) Indicate the study's design with a commonly used term in the title or the abstract	1
		(*b*) Provide in the abstract an informative and balanced summary of what was done and what was found	2
**Introduction**			
Background/rationale	2	Explain the scientific background and rationale for the investigation being reported	4-5
Objectives	3	State specific objectives, including any prespecified hypotheses	5
**Methods**			
Study design	4	Present key elements of study design early in the paper	5
Setting	5	Describe the setting, locations, and relevant dates, including periods of recruitment, exposure, follow-up, and data collection	5-8
Participants	6	(*a*) Give the eligibility criteria, and the sources and methods of selection of participants	7-8
Variables	7	Clearly define all outcomes, exposures, predictors, potential confounders, and effect modifiers. Give diagnostic criteria, if applicable	8-9
Data sources/ measurement	8*	For each variable of interest, give sources of data and details of methods of assessment (measurement). Describe comparability of assessment methods if there is more than one group	8-9 (+ codebook in repository)
Bias	9	Describe any efforts to address potential sources of bias	9
Study size	10	Explain how the study size was arrived at	7
Quantitative variables	11	Explain how quantitative variables were handled in the analyses. If applicable, describe which groupings were chosen and why	9-10
Statistical methods	12	(*a*) Describe all statistical methods, including those used to control for confounding	9-10
		(*b*) Describe any methods used to examine subgroups and interactions	9-10
		(*c*) Explain how missing data were addressed	N/A – minimal sporadic missingness

		(*d*) If applicable, describe analytical methods taking account of sampling strategy	N/A
		(*e*) Describe any sensitivity analyses	N/A
**Results**			
Participants	13*	(a) Report numbers of individuals at each stage of study—eg numbers potentially eligible, examined for eligibility, confirmed eligible, included in the study, completing follow-up, and analysed	N/A
		(b) Give reasons for non-participation at each stage	N/A
		(c) Consider use of a flow diagram	N/A
Descriptive data	14*	(a) Give characteristics of study participants (eg demographic, clinical, social) and information on exposures and potential confounders	14
		(b) Indicate number of participants with missing data for each variable of interest	N/A – Minimal sporadic missingness
Outcome data	15*	Report numbers of outcome events or summary measures	15-18
Main results	16	(*a*) Give unadjusted estimates and, if applicable, confounder-adjusted estimates and their precision (eg, 95% confidence interval). Make clear which confounders were adjusted for and why they were included	15-18
		(*b*) Report category boundaries when continuous variables were categorized	Codebook
		(*c*) If relevant, consider translating estimates of relative risk into absolute risk for a meaningful time period	N/A
Other analyses	17	Report other analyses done—eg analyses of subgroups and interactions, and sensitivity analyses	N/A
**Discussion**			
Key results	18	Summarise key results with reference to study objectives	19
Limitations	19	Discuss limitations of the study, taking into account sources of potential bias or imprecision. Discuss both direction and magnitude of any potential bias	19-20
Interpretation	20	Give a cautious overall interpretation of results considering objectives, limitations, multiplicity of analyses, results from similar studies, and other relevant evidence	20-21
Generalisability	21	Discuss the generalisability (external validity) of the study results	19-20
**Other information**			
Funding	22	Give the source of funding and the role of the funders for the present study and, if applicable, for the original study on which the present article is based	3

*Give information separately for cases and controls in case-control studies and, if applicable, for exposed and unexposed groups in cohort and cross-sectional studies.

For peer review only

Note: An Explanation and Elaboration article discusses each checklist item and gives methodological background and published examples of transparent reporting. The STROBE checklist is best used in conjunction with this article (freely available on the Web sites of PLoS Medicine at http://www.plosmedicine.org/, Annals of Internal Medicine at http://www.annals.org/, and Epidemiology at http://www.epidem.com/). Information on the STROBE Initiative is available at www.strobe-statement.org.

For peer review only

BMJ Open

Message framing to inform cancer prevention pricing interventions in the UK and US: A factorial experiment, 2019

Journal:	*BMJ Open*
Manuscript ID	bmjopen-2020-041324.R2
Article Type:	Original research
Date Submitted by the Author:	10-Dec-2020
Complete List of Authors:	Lee, Joseph; East Carolina University, Department of Health Education and Promotion, College of Health and Human Performance; University of North Carolina Lineberger Comprehensive Cancer Center, Cancer Prevention and Control<br>Cristello, Julie; Florida International University,<br>Buckton, Christina; University of Glasgow, MRC/CSO Social and Public Health Sciences Unit, Institute of Health & Wellbeing<br>Carey, Rachel; University College London, Department of Clinical, Educational & Health Psychology<br>Trucco, Elisa; Florida International University, Department of Psychology and Center for Children and Families; University of Michigan, Department of Psychiatry<br>Schenk, Paulina; University College London, Department of Clinical, Educational & Health Psychology<br>Ikegwuonu, Theresa; University of Glasgow, MRC/CSO Social and Public Health Sciences Unit, Institute of Health & Wellbeing<br>Hilton , Shona; University of Glasgow, MRC/CSO Social and Public Health Sciences Unit, Institute of Health & Wellbeing<br>Golden, SD; University of North Carolina at Chapel Hill Gillings School of Global Public Health; University of North Carolina Lineberger Comprehensive Cancer Center, Cancer Prevention and Control<br>Conway, D; University of Glasgow, School of Medicine, Dentistry, and Nursing, College of Medical Veterinary and Life Sciences
**Primary Subject Heading**:	Public health
Secondary Subject Heading:	Communication, Health policy, Oncology
Keywords:	Health policy < HEALTH SERVICES ADMINISTRATION & MANAGEMENT, PREVENTIVE MEDICINE, PUBLIC HEALTH

SCHOLARONE™
Manuscripts

I, the Submitting Author has the right to grant and does grant on behalf of all authors of the Work (as defined in the below author licence), an exclusive licence and/or a non-exclusive licence for contributions from authors who are: i) UK Crown employees; ii) where BMJ has agreed a CC-BY licence shall apply, and/or iii) in accordance with the terms applicable for US Federal Government officers or employees acting as part of their official duties; on a worldwide, perpetual, irrevocable, royalty-free basis to BMJ Publishing Group Ltd ("BMJ") its licensees and where the relevant Journal is co-owned by BMJ to the co-owners of the Journal, to publish the Work in this journal and any other BMJ products and to exploit all rights, as set out in our licence.

The Submitting Author accepts and understands that any supply made under these terms is made by BMJ to the Submitting Author unless you are acting as an employee on behalf of your employer or a postgraduate student of an affiliated institution which is paying any applicable article publishing charge ("APC") for Open Access articles. Where the Submitting Author wishes to make the Work available on an Open Access basis (and intends to pay the relevant APC), the terms of reuse of such Open Access shall be governed by a Creative Commons licence – details of these licences and which Creative Commons licence will apply to this Work are set out in our licence referred to above.

Other than as permitted in any relevant BMJ Author's Self Archiving Policies, I confirm this Work has not been accepted for publication elsewhere, is not being considered for publication elsewhere and does not duplicate material already published. I confirm all authors consent to publication of this Work and authorise the granting of this licence.

For peer review only

Public Health

For submission to: *BMJ Open*

Message framing to inform cancer prevention pricing interventions in the UK and US: A factorial experiment, 2019

Joseph G. L. Lee, PhD, MPH,[1,2*] Julie V. Cristello, MS,[3,4] Christina H. Buckton, MSc, MBA,[5] Rachel N. Carey, PhD,[6] Elisa M. Trucco, PhD,[3,4,7] Paulina M. Schenk, MSc,[6] Theresa Ikegwuonu, PhD,[5] Prof. Shona Hilton, PhD,[5] Shelley D. Golden, PhD, MPH,[2,8] Prof. David I. Conway, BDS, FDS, MPH, FFPH, PhD[9]

1. Department of Health Education and Promotion, College of Health and Human Performance, East Carolina University, Greenville, NC, USA

2. Cancer Prevention and Control, UNC Lineberger Comprehensive Cancer Center, Chapel Hill, NC, USA

3. Department of Psychology, Florida International University, Miami, FL, USA

4. Center for Children and Families, Florida International University, Miami, FL, USA

5. MRC/CSO Social and Public Health Sciences Unit, Institute of Health & Wellbeing, University of Glasgow, Glasgow, UK

6. Department of Clinical, Educational & Health Psychology, University College London, London, UK

7. Department of Psychiatry, University of Michigan, Ann Arbor, MI, USA

8. Department of Health Behavior, UNC Gillings School of Global Public Health, Chapel Hill, NC, USA

9. School of Medicine, Dentistry, and Nursing, College of Medical Veterinary and Life Sciences, University of Glasgow, Glasgow, UK

* Corresponding author: Dr. Joseph G. L. Lee, Department of Health Education and Promotion, East Carolina University, 1000 East Fifth Street Stop 529, Greenville, NC 27858; phone: +1 (252) 328-4661; fax: +1 (252) 328-1285; leejose14@ecu.edu

Key Words: public policy, policy making, prevention and control, taxes, neoplasms
Word Count: 5,191

For peer review only - http://bmjopen.bmj.com/site/about/guidelines.xhtml

Abstract (293/300 words)

Objectives: To advance understanding of how message framing can be used to maximise public support across different pricing policies for alcohol, tobacco, and sugary drinks/foods that prevent consumption of cancer-causing products.

Design: We designed a 3x4x3 randomised factorial experiment to test responses to messages with three pricing policies, four message frames, and three products.

Setting: Online survey panel (Qualtrics) in 2019.

Participants: Adults (N=1850) from the UK and USA.

Interventions: Participants randomly viewed one of 36 separate messages that varied by pricing policy (increasing taxes, getting rid of price discounts, getting rid of low-cost products), four frames, and product (alcohol, tobacco, sugary drinks/foods).

Primary and secondary outcome measures: We assessed the relationship between the message characteristics and four dependent variables. Three were related to policy support: (1) increasing taxes on the product mentioned in the message, (2) getting rid of price discounts and special offers on the product mentioned in the message, (3) getting rid of low-cost versions of the product mentioned in the message. One was related to reactance, a psychological response to having one's freedom limited.

Results: We found no effect for pricing policy in the message. Frames regarding children and reducing cancer risk moderated some outcomes, showing promise for real-world use. We found differences in support by product and reactance with greatest support and least reactance for tobacco policies, less support and more reactance for alcohol policies, and the least support and most reactance for sugary drinks/foods policies.

Conclusions: Cancer prevention efforts utilizing policy interventions can be informed by the message framing literature. Our results offer insights for cancer prevention advocacy efforts across the UK and USA and highlight that tax versus non-tax approaches to increasing the cost of cancer-causing products result in similar responses from consumers.

Article Summary

Strengths and limitations of this study

- We conducted qualitative formative work and a pilot study to inform the experimental design and stimuli.
- Our team and data represented both the UK and the USA.
- We have strong internal validity given our use of a randomised factorial design.
- Our sample size was based on a power calculation for small effect sizes.
- Testing messages in a controlled experiment likely has limited ecological validity given real-world exposure to multiple messages across multiple channels.

For peer review only - http://bmjopen.bmj.com/site/about/guidelines.xhtml

Funding

This work was supported by Cancer Research UK: BUPA Foundation Fund - International Innovation Grant (C18486/A25644), internal research funds at East Carolina University, UK Medical Research Council (MC_UU_12017/13, MC_UU_12017/15), the Chief Scientist Office of the Scottish Government Health Directorates (SPHSU13, SPHSU15), and the U.S. National Institutes of Health (K08AA023290, T32DA043449). The content is solely the responsibility of the authors and does not necessarily represent the official views of the funders.

Competing Interests

The authors report no competing interests.

Data Sharing

Data, codebooks, and interview guides are available in East Carolina University Dataverse, doi:10.15139/S3/IAKUQN, available from: https://dataverse.unc.edu/dataset.xhtml?persistentId=doi:10.15139/S3/IAKUQN

For peer review only

INTRODUCTION

One-third of the risk and burden of cancer is attributed to four common risk factors – use of tobacco, use of alcohol, unhealthy diets, and physical inactivity.[1] These individual behaviours are influenced by a complex array of factors, including people's perceptions and beliefs as well as macro-level drivers such as corporate marketing of unhealthy products including product price.[2, 3] Pricing interventions that increase the price of harmful products are an effective measure governments have to help encourage consumers to reduce the consumption of products that contribute to cancer.[4-12] There are multiple ways to increase costs of products. Many alcohol, sugary drinks/foods, and tobacco products can be subject to excise taxes, which levy a specific or proportional fee on a product that is collected as revenue by the levying government. In addition to taxation, emerging research identifies *non-tax* price-raising strategies, including policies that set a minimum product price; mitigation fees that recoup public costs from product use; minimum pack sizes that prevent very cheap product prices; minimum excise taxes that ensure a certain level of taxation; restricting sales to government stores; and bans on product price discounts and other price marketing.[9, 13-16] The World Health Organization considers such policies that raise the cost of products "best buys" for preventing and controlling non-communicable diseases.[17]

Despite strong evidence for the effectiveness of price-related policies, the implementation of such policies has been limited, due in part to industry efforts to undermine public and political support.[18-20] Indeed, substantial portions of the public remain sceptical of tax interventions and they are often described as nanny state policies or government overreach.[21-25] Despite this scepticism, support for efforts to protect children and to give everyone a comparable opportunity of achieving a healthy life are broadly supported.[26] This

For peer review only - http://bmjopen.bmj.com/site/about/guidelines.xhtml

disconnect between a lack of public support for price-related policy interventions, yet strong support for protecting people's health represents an opportunity to reframe the debate.

Public health advocates must translate potentially effective policies into ideas that resonate with public concerns.[27] The field of message framing, based in sociology and prospect theory from behavioural economics, suggests that how the information is presented changes how it is received and interpreted.[28, 29] There is good evidence showing that the framing of policies in the media or in communication campaigns can play a critical role in the adoption of policies.[25, 30-33] For example, in the US, research has suggested that mixing individual and community responsibility frames can reduce counter arguments from more conservatively-oriented members of the public, and linking other popular policies to a proposed policy can be an important strategy (e.g., a tax is connected with financing universal early childhood education or a "public health levy").[26, 34, 35] Some types of messages can perform well across the left-to-right ideological spectrum including focusing on military readiness, healthcare costs, and reducing obesity-related bullying.[36] However, messages identifying social inequities may perform poorly by invoking discriminatory stereotypes from the more advantaged groups and negative emotions from more disadvantaged groups.[26, 37, 38] In Scotland, framing minimum unit alcohol pricing policy as a public health and whole-population issue has been crucial to enabling policymakers to adopt policies.[33] Some research also suggests that showing the perceived effectiveness of a policy can have a small, positive effect on policy support.[39]

As such, framing can be an important tool in public health advocacy efforts to gain support for policies that address corporate determinants of health.[40, 41] Prior research on framing has enumerated frames used to address health-harming products.[42, 43] In addition to

For peer review only - http://bmjopen.bmj.com/site/about/guidelines.xhtml

understanding the role of the policy and the message's framing, it is important to know if there are differences in support by the type of product. Differences in support by the product could inform advocacy campaigns – especially if advocates move from a product-by-product approach to an umbrella approach for cancer-causing products.

To this end, as part of a US National Cancer Institute and Cancer Research UK sponsored Knowledge Integration Sandpit,[44] we formed a bi-national, interdisciplinary team to explore the use of framing in cancer-prevention pricing policies. We hypothesized that (**Hyp_1**) messages about non-tax pricing policies would result in greater levels of support for pricing policies than messages about tax policies given a strong distaste for taxes as a policy intervention;[23] (**Hyp_2**) pricing policies would be differentially supported based on different frames devised through a qualitative development process; and, (**Hyp_3**) tobacco-related policies would be more supported than alcohol- or sugary drinks/foods-related policies.

METHODS

Message frame development and formative interviews

This factorial experimental design contained three stages and a sample drawn from the UK and US public. In stage one, we conducted qualitative interviews with nine adults from the UK and nine adults from the US between February and May 2018. In total, we held 18 interviews, nine in each country (three members of the public, three cancer policy advocates, and three cancer survivors). We leveraged community partnerships and, in the US, student researchers' connections to churches and other organizations. We purposively sampled for diversity by age, gender, and race/ethnicity. Trained interviewers in Scotland and North Carolina conducted interviews in their respective countries using a semi-structured interview guide.

For peer review only - http://bmjopen.bmj.com/site/about/guidelines.xhtml

Briefly, we asked participants about their initial thoughts, advantages/disadvantages, and benefits/harms of four pricing policies (minimum prices, getting rid of coupons or discounts, adding taxes, and mitigation fees). We then asked about level of support for policies targeting specific products: alcohol, sugary drinks/foods, and tobacco. Finally, we asked what arguments for a pricing policy would be persuasive. All interviews were professionally transcribed verbatim, and transcriptions checked by the original interviewer. Transcripts were entered into QSR International's NVivo 12 qualitative data analysis software for thematic analysis.[45] Two researchers read all transcripts in depth and carried out initial coding following the interview guide structure. We then inductively identified recurrent themes for each price policy and compared similarities and differences in views between price policies, products, and countries.

In stage two, we used the results from the formative qualitative interviews and the published literature[22, 26, 33, 43] to develop nine potential frames and iteratively refined them for clarity and readability (a description of the potential frames is available in our repository[46]). For example, based on suggestions from the Robert Wood Johnson Foundation,[26] we developed a frame highlighting individual and community responsibility: "We can all play a role in making healthier communities. Everyone can contribute to this by making healthy choices regarding their own alcohol consumption, but also by supporting increasing taxes on alcohol." We then pilot-tested the frames with 106 UK and US adults in an online survey panel provided by Qualtrics Research Services, 30th October-1st November 2018. Participants reported how well they understood the potential frames on a 3-point scale (1=understood very well, 2=understood fairly well, 3=did not understand well), described each in their own words and ranked the nine frames from most to least compelling. To select the frames for our main experiment, we computed the self-reported mean comprehensibility and the average

For peer review only

For peer review only - http://bmjopen.bmj.com/site/about/guidelines.xhtml

ranking for each frame, and double coded (on a 3-point scale) whether participants accurately described the frame. In this step we did not manipulate product or policies; all messages were about alcohol taxes.

In the third and final stage, we selected four frames for an experiment. The four frames (Table 1) were adapted to each of three products (alcohol, sugary drinks/foods, tobacco) and each of three policies (taxes, minimum pricing, getting rid of discounting). We created 36 messages specific to product, policy, and the four frames about how the policy intervention would (1) reduce strain to healthcare, (2) protect children, (3) prevent cancer, and (4) bring consumption of the product down. All messages are provided in our linked Dataverse repository.

Table 1. Example frames used in experiment

**Frame Number (short description)**	**Example frame for alcohol product and tax policy**
F_1 (reduce strain on healthcare)	The problems caused by alcohol misuse, such as crime, injuries and addiction, are costly. Increasing taxes on alcohol will reduce the financial strain on our overburdened health care system.
F_2 (protect children)	Alcohol misuse can hurt children's health and wellbeing - both directly (through children drinking alcohol), and indirectly (through adults' misuse of alcohol impacting children). We can protect children by increasing taxes on alcohol.
F_3 (prevent cancer)	Drinking less alcohol can reduce the risk of cancer. Increasing taxes on alcohol is an important way to prevent cancer in our communities.
F_4 (bring consumption down)	Drinking less alcohol can reduce the risk of stroke, high blood pressure, obesity, liver disease, and mental health problems. Increasing taxes on alcohol is an important way to bring drinking levels down.

Experimental design and implementation

We conducted an *a-priori* power analysis for the 3x4x3 factorial design using G*Power 3.1[47] with power (1 - β) set at 0.80 and α=.05, two-tailed, for a small effect size (Cohen's f =0.10) given prior findings examining similar constructs.[35, 36] The power analysis indicated that a sample of 1745 would be needed to detect these small effects. To recruit participants, we engaged Qualtrics Research Services, an online panel service; this approach shows evidence of

For peer review only - http://bmjopen.bmj.com/site/about/guidelines.xhtml

generalisability for experiments.[48, 49] We used quota sampling to include 50% of participants from the UK and 50% from the US, equal representation by assigned birth sex, a minimum of 20% of participants with fewer than four years of post-secondary education, and a minimum of 20% of participants reporting having smoked 100 or more cigarettes in one's life. Qualtrics provided each participant "points" in exchange for completing the survey that could be redeemed for incentives such as gift cards. Qualtrics successfully recruited and provided data from 1,850 participants. In this between-subjects experimental factorial design, each participant was randomly assigned to view one of the 36 messages using Qualtrics's advanced block programming. Table 2 shows the experiment design with blue text for the cancer product and red text for the policy.

Table 2. Frame x products x policy factorial design

		Policy		
		Tax	Getting Rid of Discounting	Min. Price
Cancer Product	Alcohol	F_1 A T	F_1 A D	F_1 A P
		F_2 A T	F_2 A D	F_2 A P
		F_3 A T	F_3 A D	F_3 A P
		F_4 A T	F_4 A D	F_4 A P
	Sugary Drinks/Foods	F_1 S T	F_1 S D	F_1 S P
		F_2 S T	F_2 S D	F_2 S P
		F_3 S T	F_3 S D	F_3 S P
		F_4 S T	F_4 S D	F_4 S P
	Tobacco	F_1 T T	F_1 T D	F_1 T P
		F_2 T T	F_2 T D	F_2 T P
		F_3 T T	F_3 T D	F_3 T P
		F_4 T T	F_4 T D	F_4 T P

After brief screening and demographic questions, we provided the following prompt: "On the next screen, we will show you a message about a public health policy and a product. After you read the message, we'll ask you questions about what you think about policies about that product. PLEASE READ THE MESSAGE ABOUT THE PRODUCT ON THE NEXT

For peer review only - http://bmjopen.bmj.com/site/about/guidelines.xhtml

SCREEN. You will not be able to see it again." The participant then viewed one of the 36 messages.

Measures

To assess the impact of the different products, policies, and frames in our messages, we used four dependent variables. Following prior work by Niederdeppe,[35] we first measured policy support with three questions: "How much do you oppose or support the following?" i) Increasing taxes on the product mentioned in the message, ii) Getting rid of price discounts and special offers on the product mentioned in the message, and iii) Getting rid of low-cost versions of the product mentioned in the message. There were five response options ranging from Strongly Oppose (1) to Strongly Support (5) with a neutral point. Each participant answered all three questions regardless of the policy invoked in their message frame. We also measured reactance, a psychological response to having one's freedom limited,[50] to the message using six items adapted from Hall,[51] e.g., "The product mentioned is legal, so the government should stop interfering with it." There were five response options ranging from Strongly Disagree (1) to Strongly Agree (5) with a neutral point, which we averaged into a scale. These items exhibited good internal consistency in our data (McDonald's omega=0.87) and could not be improved by removing any item.[52]

We also measured potential covariates and factors including country (UK, US), and use of alcohol, sugary beverages, and cigarettes in the last 30 days (options: every day, some days, not at all). Full details of measures are available in our repository.

Analysis of Experiment

For peer review only - http://bmjopen.bmj.com/site/about/guidelines.xhtml

We assessed whether the: (1) type of policy, (2) product, or (3) frame used in a message was associated support for each type of policy and reactance, as well as interactions among these main predictors. We also assessed interactions between covariates (sex, age, country) and main predictors; if they were significant, those covariates were analysed as an additional factor as was the case for country in two of the models. All main effects and interactions were analysed using a 3x4x3 ANOVA. In two of the models, where country was significant, a 2x3x4x3 ANOVA was conducted. We chose ANOVA over a regression framework as an ANOVA approach provides equivalent results to linear regression with dummy-coded predictors[53] and we did not wish to compare results to a single reference category. When main effects or interactions were significant, we conducted pairwise comparisons using Fisher's least significant differences test. We used a traditional threshold of statistical significance, $p<0.05$ and used two tailed tests. We used SPSS v.25 (IBM, Chicago, IL).

Research undertaken in this project was reviewed by the East Carolina University and Medical Center Institutional Review Board (#17-002904) and University of Glasgow College of Medical, Veterinary & Life Sciences Ethics Committee (#200170074). Verbal consent was obtained from all interviewees and online survey experiment participants received study information and were asked to continue to the study if they agreed to participate.

Patient and Public Involvement

Patients and the public were not involved in this research.

RESULTS

Formative interviews

We completed formative interviews with eighteen participants, nine in the UK and nine in the US. Interviewees were between ages 24 and 69 years (average age 48), and cancer

For peer review only - http://bmjopen.bmj.com/site/about/guidelines.xhtml

survivors had a range of cancer experiences including prostate, lung, breast, head and neck, and skin cancers. Interviews lasted between twenty minutes and one hour depending on the interviewees' interest in the subject area. In general, interviewees were more supportive of price policies for tobacco containing commodities than either alcohol or sugary drinks/foods (Table 3).

Table 3: Interviewees' overall support for price policies in general by product type, country, and interviewee type, N=18

Product	UK interviewees									US interviewees									Mean
	Advocates			Survivors			Public			Advocates			Survivors			Public			
Tobacco	9	10	8	8	9	10	1	9	9	8	9	8	8	5	8	7	8	8	7.9
Alcohol	9	10	4	3	7	7	1	5	6	7	6	4	3	5	5	4	8	6	5.6
Sugary Drinks/Foods	10	10	3	8	4	8	3	4	9	5	3	2	7	5	2	5	6	4	5.4

Note: 1=not supportive, 10=very supportive.

During the interviews it became apparent that participants found it difficult to differentiate between a hypothecated tax and a mitigation fee. The team discussed this issue and decided to drop mitigation fee as a policy in the main experiment. A number of emergent themes were common to all three pricing policies (Table 4). Specifically, participants thought that all of the policies would have an impact on purchasing behaviour with a concomitant health benefit due to reduced consumption, particularly for young people and those on low income. Participants also thought they could have an indirect benefit by drawing attention to the product and the level of potential health harm. For example, a policy advocate and cancer survivor said, respectively:

'*…it would reduce the excessive consumption of these products, it would control the quantities in which these products are consumed, and it would mean the consumer… is more aware of their purchasing of the products.*' (Policy advocate)

For peer review only

For peer review only - http://bmjopen.bmj.com/site/about/guidelines.xhtml

'The biggest benefit is that it will make the person aware – some people drink and eat things they're not aware of, they don't look into it – and if they see that there's a higher tax, and they start recognising that, they're going to find out why and it might change their behaviour.' (Cancer survivor)

[Table 4 appears on next page.]

For peer review only

For peer review only - http://bmjopen.bmj.com/site/about/guidelines.xhtml

Table 4. Themes identified in qualitative interviews

Common for all 3 pricing policies	Specific to minimum price	Specific to getting rid of discounts	Specific to tax
**Supportive arguments/ advantages**			
• Health benefit<br>• Greatest benefit for young<br>• Greatest benefit for low SES<br>• Will affect purchasing behaviour<br>• Will highlight real cost of consumption/health harms<br>• More supportive where there is clear evidence of cancer links	• A deterrent or nudge<br>• Consistency and fairness<br>• Targets most harmful consumption<br>• There is evidence to support it	• Price promotions most strongly influence purchasing<br>• Promotions encourage over consumption<br>• Will protect industry and retailer profits	• Could prompt reformulation<br>• Could be a form of health insurance<br>• Source of public revenue to fund social policy programmes/ health services<br>• An effective way of moderating behaviour
**Unsupportive arguments/ disadvantages**			
• Effectiveness will depend on individual response<br>• Needs to be prohibitively high to change consumption<br>• Unfair on responsible consumers<br>• Addicts will pay more<br>• Could create a black market (US specific)<br>• Regressive<br>• Bad for retailers/ producers<br>• Simplistic – not enough	• Contravenes EU law<br>• Public do not understand/ will not support it<br>• Revenue raised goes to industry<br>• Too specific for food<br>• Will not impact population level drinking	• Lost opportunity to apply to healthy foods<br>• Will push prices up across the board<br>• Inappropriate to interfere in pricing	• Nanny statist - interference in freedom of choice<br>• Political cost – unpopular<br>• Reformulation could prompt increased consumption<br>• Negative unintended consequences - substitutes may be equally unhealthy<br>• Will not work if funds are not ring-fenced

For peer review only - http://bmjopen.bmj.com/site/about/guidelines.xhtml

Some key disadvantages were also highlighted across all policies: (1) that their effectiveness would depend on the scale of the price increase and how individuals responded; (2) that they are potentially regressive; and, (3) that they are unfair to responsible consumers and to those who are dependent on these substances. Participants were especially concerned about the impact on individuals dependent on these products with limited resources, particularly in relation to alcohol consumption. Yet, this concern also applied to tobacco and sugary drinks/foods, which were both deemed to have addictive qualities. Quotes illustrating these disadvantages include:

> *'The effect on health does depend on what decisions people make in response to it. And whether it follows what we expect. And does it reduce purchase of those products, does it push people to other products or not?'* (Policy advocate)

> *'I think they [people with limited incomes] would then be more likely to stop buying food, to stop heating their house, or to stop paying their rent or, you know, whatever, in order to prioritize the alcohol consumption.'* (General public)

There were a number of unique themes arising for each price policy (Table 3). For example, although discounts and price promotions were thought to be the strongest influencer of purchasing behaviour and to encourage over consumption, interviewees thought they should be refocused on healthier foods and drinks rather than replaced. For minimum unit price policies specifically, interviewees noted that an advantage of such policies is that they target the most harmful patterns of consumption, i.e., the highest levels of consumption which can result in anti-social behaviour. Taxation alone prompted interviewees to talk about nanny state arguments and inappropriate government intervention in the market. This response was more marked in the UK

For peer review only - http://bmjopen.bmj.com/site/about/guidelines.xhtml

332 than the US and might be explained by the fact that the timing of the interviews coincided with the implementation of the UK Soft Drinks Industry Levy ("Sugar Tax") in April 2018.

The frames that interviewees thought would be most persuasive and result in increased support for price policies could be summarised as those that: (1) draw a very clear link to potential health harm from consuming the product; (2) focus on a positive health impact arising from the policy, particularly protection of children; (3) emphasise the impact on health services in terms of reduced costs and the potential to generate revenue for reinvestment; and, (4) make the policies meaningful in terms of the anticipated benefit for individuals, in straight forward language. Less convincing frames were those that: (1) employ threats or scare tactics; (2) seem condescending or nanny statist; (3) use jargon or statistics; and, (4) set unrealistic expectations of behaviour change for individuals. We used these findings to inform the experimental stimuli, results for which are presented next.

Main experimental effects

Participants were 1,850 adults (ages 18-86, M_{age}=38.56, SD=13.56). The sample was evenly split across the countries of interest (50.1% from the UK) and sex assigned at birth (50.2% female). Within the sample, 34.7% graduated from college or university, 26.4% attended some college or university, 28.9% had graduated or completed secondary or high school, and the remainder (7.6%) attended some high school or less. Table 5 shows the pattern of results.

[Table 5 appears on the next page.]

For peer review only - http://bmjopen.bmj.com/site/about/guidelines.xhtml

Table 5. Summary of significance direction of main effects and interactions between factors and dependent variables, 2019, N=1,850, UK and US adults

**Factors**	**Dependent variables**			
	**Support for raising taxes**	**Support for getting rid of price discounts and special offers**	**Support for getting rid of low-cost version of product**	**Psychological reactance**
Policy (taxes, minimum pricing, getting rid of discounting)	• Main: NS<br>• Interactions: NS	• Main: NS<br>• Interactions: NS	• Main: NS<br>• Interactions: NS	• Main: NS<br>• Interactions: NS
Frame (1. reduce strain to healthcare, 2. protect children, 3. prevent cancer, and 4. bring consumption of the product down)	• Main: NS<br>• Interactions: NS	• Main: Higher support with Frame 2 (Children) and 3 (Prevent Cancer) than 4 (Reduce Use)<br>• Interaction: NS	• Main: NS<br>• Interaction: For participants receiving Frame 3 (Prevent Cancer) support was higher for minimum pricing policy in message than for tax policy in message<br>• For participants receiving Frame 4 (Reduce Use) differences in support by policy in message	• Main: NS<br>• Interaction: Frame 2 (Children) lowered reactance for UK participants more than US participants
Product (alcohol, sugary drink/food, tobacco)	• Main: Tobacco: Highest; Alcohol: Lower; Sugar: Lowest;<br>• Interaction: Tobacco higher for UK than US	• Main: Tobacco: Highest; Alcohol: Lower; Sugar: Lowest<br>• Interaction: NS	• Main: Tobacco: Highest; Alcohol: Lower; Sugar: Lowest<br>• Interaction: NS	• Main: Sugar: Highest; Alcohol: Lower; Tobacco: Lowest<br>• Interaction: Sugar and Tobacco lower for UK than US
Country (UK, USA)†	• Main: UK: Higher; US: Lower<br>• Interaction: NS	• Main: NS<br>• Interaction: NS	• Main: NS<br>• Interaction: NS	• Main: US: Higher; UK: Lower<br>• Interaction: NS

For peer review only - http://bmjopen.bmj.com/site/about/guidelines.xhtml

353 Note: Sugar = Sugary Drinks/Foods, NS=not statistically significant; † Covariate assessed as a factor given significant interactions with other factors; interactions assessed were between covariates (sex, age, country) and main predictors (policy, frame, product; if they were significant, those covariates were analysed as an additional factor as was the case for country in two of the models

Dependent variable 1: Support for increasing taxes on the product mentioned in the message

For the first outcome, there was not a significant main effect for policy (i.e., taxes, minimum pricing, getting rid of discounting) or frame (i.e., reduce strain to healthcare, protect children, prevent cancer, and bring consumption of the product down). There was a significant main effect for product (F[2, 1767]=45.71, p<0.001) and country (F[1, 1767]=26.63, p<0.001). Pairwise comparisons for product revealed significant differences across all three products (M_{sugar}=2.76, SD=1.26; $M_{alcohol}$=3.07, SD=1.30; $M_{tobacco}$=3.47; SD=1.37); participants receiving a message regarding sugary drinks/foods had the lowest and participants receiving a message regarding tobacco endorsed the highest levels of support for increasing taxes on the product. Pairwise comparisons for country revealed that participants from the UK (M_{UK}=3.257, SD=1.33) endorsed higher levels of support for increasing taxes compared to participants from the US (M_{US}=2.937, SD=1.34). There was also a significant two-way interaction: product x country, F(2, 1767)=5.67, p<0.01. Specifically, among participants receiving a sugary drinks/foods product message, those from the UK endorsed higher levels of support for increasing taxes on the product (M_{UK}=3.08, SD=1.28) compared to US participants (M_{US}=2.51, SD=1.19). Similarly, among participants receiving a tobacco product message, those from the UK endorsed higher levels of support for increasing taxes on the product (M_{UK}=3.69, SD=1.32) compared to US participants (M_{US}=3.24, SD=1.38). Three-way interactions were not significant.

Dependent variable 2: Support for getting rid of price discounts and special offers on the product mentioned in the message

For peer review only - http://bmjopen.bmj.com/site/about/guidelines.xhtml

For the second outcome, there was not a significant main effect for policy (i.e., taxes, minimum pricing, getting rid of discounting). There was a significant main effect for frame (F[3, 1803]=2.62, p<0.05) and message product (F[2, 1803]=53.80, p <0.001). Participants receiving frames 2 (protect children, M_{frame2}=3.25, SD=1.37) or 3 (prevent cancer, M_{frame3}=3.24, SD=1.35) endorsed higher levels of support for getting rid of price discounts and special offers than participants who received frame 4 (bring consumption down, M_{frame4}=3.04, SD=1.36). Pairwise comparisons for product revealed significant differences across all three products ($M_{sugar=}$2.85, SD=1.28; $M_{alcohol}$=3.05, SD=1.34; $M_{tobacco}$=3.59; SD=1.35); participants receiving a message regarding sugary drinks/foods and participants receiving a message regarding tobacco endorsed the lowest and highest levels of support for getting rid of price discounts and special offers on the product, respectively. There were no significant two-way or three-way interactions.

Dependent variable 3: Support for getting rid of low-cost versions of the product mentioned in the message

For the third outcome, there was not a significant main effect for policy (i.e., taxes, minimum pricing, getting rid of discounting) or frame (i.e., reduce strain to healthcare, protect children, prevent cancer, and bring consumption of the product down). There was a significant main effect for message product (F(2, 1802)=60.34, p<0.001). Pairwise comparisons revealed significant differences across all three products ($M_{sugar=}$2.82, SD=1.26; $M_{alcohol}$=3.08, SD=1.29; $M_{tobacco}$=3.60; SD=1.34), such that participants receiving a message regarding sugary drinks/foods and participants receiving a message regarding tobacco endorsed the lowest and highest levels of support for getting rid of low-cost versions of the product, respectively. There was also a significant two-way interaction: frame x policy, F(6, 1802)=2.24, p<0.05. Specifically, among participants receiving frame 3 (prevent cancer), participants who received

For peer review only - http://bmjopen.bmj.com/site/about/guidelines.xhtml

the policy related to minimum pricing endorsed higher levels of support for getting rid of low-cost versions of the product ($M_{minprice}$=3.36, SD=1.28) compared to those receiving the policy related to increasing taxes (M_{tax}=3.05, SD=1.38). Additionally, among participants receiving frame 4 (bring consumption down), there were significant differences in support for getting rid of low-cost products between participants who received policies related to getting rid of discounting ($M_{nodiscounting}$=3.23, SD=1.30) and minimum pricing ($M_{minprice}$=3.16, SD=1.37), and participants who received the policy related to increasing taxes (M_{tax}=2.76, SD=1.29). There were no significant three-way interactions.

Dependent variable 4: Reactance

For the last outcome, there was not a significant main effect for policy or frame on reactance (i.e., the extent to which one perceives their freedoms to be limited). There was a significant main effect for message product (F[2, 1769]=30.74, p<0.001) and country (F[1, 1769]=7.21, p<0.01). Pairwise comparisons for product revealed significant differences across all three products ($M_{tobacco}$=2.72; SD=0.99; $M_{alcohol}$=2.92, SD=0.95; $M_{sugar=}$3.14, SD=0.93), such that participants receiving a message regarding tobacco and participants receiving a message regarding sugary drinks/foods, indicated the lowest and highest levels of reactance, respectively. Pairwise comparisons for country revealed that participants from the US (M_{US}=2.99, SD=0.97) endorsed higher levels of reactance compared to participants from the UK (M_{UK}=2.87, SD=0.97). There were two significant two-way interactions: product x country, F(2, 1769)=4.09, p<0.05 and frame x country F(3, 1769)=2.84, p<0.05. Specifically, among those that received a message regarding tobacco, participants from the US endorsed significantly higher levels of reactance (M_{US}=2.838, SD=0.98) compared to participants from the UK (M_{UK}=2.606, SD=0.98). Similarly, among those receiving a message regarding sugary drinks/foods, participants from the US

For peer review only

endorsed significantly higher levels of reactance (M_{US}=3.24, SD=0.91) compared to participants from the UK (M_{UK}=3.05, SD=0.93). Among those who received frame 2 (protect children), participants from the UK endorsed lower levels of reactance (M_{UK}=2.71, SD=0.97) than those from the US (M_{US}=3.01, SD=0.93).

Lastly, there was also a significant three-way interaction: product x policy x country, (F[4, 1769]=2.97, p<0.05). Among those receiving a message regarding alcohol, those from the UK endorsed significantly higher levels of reactance (M_{UK}=3.05, SD=0.92) compared to participants from the US (M_{US}=2.78, SD=0.94) receiving the getting rid of discounting policy. In contrast, among those receiving a message regarding sugary drinks/foods, those from the US endorsed significantly higher levels of reactance (M_{US}=3.39, SD=0.92) compared to participants from the UK (M_{UK}=3.07, SD=0.95) for those receiving the policy related to increasing taxes. Among those receiving a message regarding tobacco, those from the US endorsed significantly higher levels of reactance (M_{US}=2.93, SD=1.05) compared to participants from the UK (M_{UK}=2.48, SD=0.95) receiving the getting rid of discounting policy.

DISCUSSION

Principal findings

This research aimed to explore whether the specific content of a message about cancer-prevention price policies was associated with higher public support and lower reactance for those policies. We had three main hypotheses. First, we hypothesized that messages about non-tax pricing policies would result in more support than messages about tax policies. Our results did not provide evidence for this hypothesis: Not one of the three policies tested (taxes, minimum pricing, and getting rid of discounts) showed a statistically significant impact on support in our experiment. Regarding our second hypothesis, that support for a particular pricing policy would

For peer review only - http://bmjopen.bmj.com/site/about/guidelines.xhtml

change based on the framing of the message, we found some evidence. Specifically, of the frames we tested, which were selected based on their performance in pilot testing, our findings indicate that frames relating to children show promise. We found evidence to support our third hypothesis that messages about tobacco products would result in more support for raising the price of products than messages about alcohol or sugary drinks/foods products. Product in the message also changed reactance, which was highest for sugary drinks/foods and lowest for tobacco. Finally, we found statistically significant differences in responses to messages by country (e.g., support for alcohol pricing interventions was lower in the US than in the UK).

Strengths and weaknesses

The strong internal validity from the experimental design, qualitative formative work, and international scope of this research must be balanced against limitations. First, this study used an online convenience sample from a panel provider service, Qualtrics, which may limit its external validity given the participants are not representative of their respective countries, particularly in tobacco use and educational attainment. However, prior research indicates that convenience samples like ours generalize well when used in experimental studies rather than studies of prevalence.[48] Second, we assessed the impact of a single exposure of a message displayed on a screen. This does not reflect the real-world environment where messages about policies may be delivered from multiple sources, repeated, and seen on multiple communication channels. Additionally, a limitation to survey research is that participants can interpret response scales differently which can impact data and interpretations. Thus, participants were provided with a wider range of responses (e.g., 5-point Likert scales) and category labels to minimize these variations. Third, further work should explore equity implications of our messaging

For peer review only - http://bmjopen.bmj.com/site/about/guidelines.xhtml

approaches – especially given growing evidence regarding the pro-equity effect of pricing interventions.[54]

Results in context

The process of policy development as well as policy making decisions are ultimately taken by politicians on behalf of their constituents (the public); in this context, the scientific evidence- and value-bases play important roles.[55] While the evidence-base for different types and targets of price policies on behaviour is rapidly expanding,[56-61] the difficulty of increasing public support and consequently political support for pricing interventions has received less attention. The relatively low levels of public support have previously been highlighted.[22, 62] Our findings provide further evidence to suggest limited support among the public for pricing interventions overall, given relatively low endorsements of support on our support dependent variables. However, given the mean scores, the average participant was also not that opposed. Furthermore, a policy message that specifically referenced a particular type of price policy did not result in more support for that type of price policy compared to other types.

Prior research has examined the role of framing in public health efforts, and our findings demonstrate ways to best utilize media advocacy approaches[63] by minimizing reactance from the public and promoting support of evidence-based policies. Specifically, our frames were all selected based on the prior literature and their performance in formative work. Particular value may come from framing messages around the protection of children. This is consistent with previous work.[26] Previous work has also shown that the product under consideration influences policy support.[64] Our experimental findings offer further evidence of this, showing that participants rated support of pricing interventions highest on messages for tobacco products, followed by alcohol, and lastly by sugary drinks/foods. Finally, our findings highlight some

For peer review only - http://bmjopen.bmj.com/site/about/guidelines.xhtml

notable differences by country, which may reflect more general differences in acceptance of policies, healthcare systems, and political developments at the time of our study (e.g., 'Brexit').

Implications for Public Health Policy and Practice

Our findings have several implications for messaging about pricing policies and for policy research, particularly with the aim of garnering support (across stakeholder groups from public, civil society, media, and politicians) to develop and implement price policies. First, the evidence of differences by product may indicate that efforts to "denormalize" tobacco products may have yet to translate to alcohol and sugary drinks/foods efforts. Policy advocates should consider if lessons learned from tobacco control can and should be applied to alcohol and sugary drinks/foods. It is worth highlighting that denormalization can present the risk of increasing stigma, which has been a criticism in the area of tobacco.[65] Second, our findings suggest the importance of bridging the research-practice gap between the literature on framing and advocacy organizations' messaging. Frames that include a focus on protecting children seem to show particular promise; however, our four frames showed few significant difference in their overall performance. This is likely because we used a robust pilot testing process to develop the frames, and we only selected the best performing frames for use in the main experiment. Practitioners should consider use of the four frames tested here. Third, practitioners should consider lessons learned from other countries, which may help with identifying particular challenges and successes regarding cancer prevention.

Conclusions

In our online experiment with participants from the UK and US, we found no significant differences in price policy message support or reactance based on the type of proposed policy, suggesting advocates can recommend policies that raise prices through both tax and non-tax

For peer review only - http://bmjopen.bmj.com/site/about/guidelines.xhtml

means. Results also indicate that framing messages focused on protecting children had increased support and lowered reactance in some combinations of experimental stimuli. Public health policy advocates should consider using frames like those we tested in ongoing efforts to develop, adopt, and implement price policies that reduce use of cancer-causing products. This study also indicates that lessons learned from efforts to build public support for addressing tobacco use may be needed to successfully address alcohol and sugary drinks/products, since support for pricing policies for these products were generally lower than for tobacco price policies. Public health practitioners and advocates should consider using tested messages when working to advance pricing policies that reduce consumption of harmful products marketed by powerful corporations.

Author Contributions

JGLL, RNC, EMT, DIC conceived the study. JGLL, CHB, RNC, EMT, TI, SH, SDG, and DIC designed the study. JVC (quantitative) and CHB (qualitative) conducted the data analysis with input from EMT and SH, respectively. PMS conducted analyses of the pilot study with input from RNC. JGLL, CHB, RNC, EMT, PMS, SDG, and DIC developed the stimuli. JGLL led the manuscipt drafting; all authors provided critical input on the interpretation of the results, writing of the manuscript, and approved the final version.

Human Participant Protections

Research undertaken in this project was reviewed by the East Carolina University and Medical Center Institutional Review Board (#17-002904) and University of Glasgow College of Medical, Veterinary & Life Sciences Ethics Administration Committee (#200170074).

Acknowledgements

We thank Cancer Research UK and the US National Cancer Institute Health Behaviours Research Branch for the opportunity to participate in their jointly hosted 2017 “sandpit” workshop. We thank Israel M. Mendez for his help with the data collection and project management in the US. An earlier version of this paper was presented in a dissemination event held at the Royal Institution in London in November 2019.

For peer review only - http://bmjopen.bmj.com/site/about/guidelines.xhtml

REFERENCES

[1] Parkin DM, Boyd L, Walker LC. 16. The fraction of cancer attributable to lifestyle and environmental factors in the UK in 2010. *Br J Cancer* 2011;**105 Suppl 2**:S77-81.

[2] Madureira Lima J, Galea S. Corporate practices and health: a framework and mechanisms. *Global Health* 2018;**14**(1):21.

[3] McKee M, Stuckler D. Responding to the Corporate and Commercial Determinants of Health. *Am J Public Health* 2018:e1-e4.

[4] Contreary KA, Chattopadhyay SK, Hopkins DP*, et al.* Economic impact of tobacco price increases through taxation: a Community Guide systematic review. *Am J Prev Med* 2015;**49**(5):800-808.

[5] IARC. Effectiveness of tax and price policies for tobacco control. *IARC Handbooks of Cancer Prevention*. Lyon, France: International Agency for Research on Cancer 2011.

[6] Wagenaar AC, Salois MJ, Komro KA. Effects of beverage alcohol price and tax levels on drinking: a meta-analysis of 1003 estimates from 112 studies. *Addiction* 2009;**104**(2):179-190.

[7] Powell LM, Chriqui JF, Khan T*, et al.* Assessing the potential effectiveness of food and beverage taxes and subsidies for improving public health: a systematic review of prices, demand and body weight outcomes. *Obes Rev* 2013;**14**(2):110-128.

[8] Burton R, Henn C, Lavoie D*, et al.* A rapid evidence review of the effectiveness and cost-effectiveness of alcohol control policies: an English perspective. *Lancet* 2017;**389**(10078):1558-1580.

[9] Boniface S, Scannell JW, Marlow S. Evidence for the effectiveness of minimum pricing of alcohol: a systematic review and assessment using the Bradford Hill criteria for causality. *BMJ Open* 2017;**7**(5):e013497.

[10] Eyles H, Ni Mhurchu C, Nghiem N*, et al.* Food pricing strategies, population diets, and non-communicable disease: a systematic review of simulation studies. *PLoS Med* 2012;**9**(12):e1001353.

[11] Afshin A, Penalvo JL, Del Gobbo L*, et al.* The prospective impact of food pricing on improving dietary consumption: A systematic review and meta-analysis. *PLoS One* 2017;**12**(3):e0172277.

[12] Teng AM, Jones AC, Mizdrak A*, et al.* Impact of sugar-sweetened beverage taxes on purchases and dietary intake: Systematic review and meta-analysis. *Obes Rev* 2019;**20**(9):1187-1204.

[13] Golden SD, Farrelly MC, Luke DA*, et al.* Comparing projected impacts of cigarette floor price and excise tax policies on socioeconomic disparities in smoking. *Tob Control* 2016;**25**(Suppl 1):i60-i66.

[14] Golden SD, Smith MH, Feighery EC*, et al.* Beyond excise taxes: a systematic review of literature on non-tax policy approaches to raising tobacco product prices. *Tob Control* 2016;**25**(4):377-385.

[15] Hiscock R, Augustin NH, Branston JR*, et al.* Standardised packaging, minimum excise tax, and RYO focussed tax rise implications for UK tobacco pricing. *PLoS One* 2020;**15**(2):e0228069.

[16] Kerr WC, Williams E, Greenfield TK. Analysis of price changes in Washington following the 2012 liquor privatization. *Alcohol Alcohol* 2015;**50**(6):654-660.

For peer review only

For peer review only - http://bmjopen.bmj.com/site/about/guidelines.xhtml

[17] World Health Organization. Tackling NCDs: 'Best Buys' and Other Recommended Interventions for the Prevention and Control of Noncommunicable Diseases. Geneva: World Health Organization 2017.

[18] Berg CJ, Solomon M, Barkley A*, et al.* Tobacco taxes in the Southeastern US States: views from former legislators. *Health Behav Policy Rev* 2015;**2**(5):333-342.

[19] Katikireddi SV, Hilton S. How did policy actors use mass media to influence the Scottish alcohol minimum unit pricing debate? Comparative analysis of newspapers, evidence submissions and interviews. *Drugs (Abingdon, England)* 2015;**22**(2):125-134.

[20] Holmes CB, King BA, Babb SD. Stuck in neutral: stalled progress in statewide comprehensive smoke-free laws and cigarette excise taxes, United States, 2000-2014. *Prev Chronic Dis* 2016;**13**:E80.

[21] Stockwell T. Minimum unit pricing for alcohol: the most cost-effective of cancer prevention strategies? *Expert Rev Anticancer Ther* 2017;**17**(11):981-983.

[22] Barry CL, Niederdeppe J, Gollust SE. Taxes on sugar-sweetened beverages: results from a 2011 national public opinion survey. *Am J Prev Med* 2013;**44**(2):158-163.

[23] Petrescu DC, Hollands GJ, Couturier DL*, et al.* Public acceptability in the UK and USA of nudging to reduce obesity: the example of reducing sugar-sweetened beverages consumption. *PLoS One* 2016;**11**(6):e0155995.

[24] Eykelenboom M, van Stralen MM, Olthof MR*, et al.* Political and public acceptability of a sugar-sweetened beverages tax: a mixed-method systematic review and meta-analysis. *Int J Behav Nutr Phys Act* 2019;**16**(1):78.

[25] Weishaar H, Dorfman L, Freudenberg N*, et al.* Why media representations of corporations matter for public health policy: a scoping review. *BMC Public Health* 2016;**16**:899.

[26] RWJF. A new way to talk about the social determinants of health. 2010. Accessed December 10, 2020. Available from: https://www.rwjf.org/en/library/research/2010/01/a-new-way-to-talk-about-the-social-determinants-of-health.html

[27] Smith K. Beyond Evidence Based Policy in Public Health: The Interplay of Ideas. New York, NY: Palgrave 2013.

[28] Goffman E. Frame analysis: An Essay on the Organization of Experience. Cambridge, MA: Harvard University Press 1974.

[29] Tversky A, Kahneman D. The framing of decisions and the psychology of choice. *Science* 1981;**211**(4481):453-458.

[30] Benford RD, Snow DA. Framing processes and social movements: an overview and assessment. *Annu Rev Sociol* 2000;**26**(1):611-639.

[31] Cacciatore MA, Scheufele DA, Iyengar S. The end of framing as we know it… and the future of media effects. *Mass Commun Soc* 2016;**19**(1):7-23.

[32] Koon AD, Hawkins B, Mayhew SH. Framing and the health policy process: a scoping review. *Health Policy Plan* 2016;**31**(6):801-816.

[33] Katikireddi SV, Bond L, Hilton S. Changing policy framing as a deliberate strategy for public health advocacy: a qualitative policy case study of minimum unit pricing of alcohol. *Milbank Q* 2014;**92**(2):250-283.

[34] Purtle J, Langellier B, Le-Scherban F. A case study of the Philadelphia sugar-sweetened beverage tax policymaking process: Implications for policy development and advocacy. *J Public Health Manag Pract* 2018;**24**(1):4-8.

For peer review only

[35] Niederdeppe J, Roh S, Shapiro MA. Acknowledging individual responsibility while emphasizing social determinants in narratives to promote obesity-reducing public policy: a randomized experiment. *PLoS One* 2015;**10**(2):e0117565.

[36] Gollust SE, Niederdeppe J, Barry CL. Framing the consequences of childhood obesity to increase public support for obesity prevention policy. *Am J Public Health* 2013;**103**(11):e96-102.

[37] Landrine H, Corral I. Targeting cancer information to African Americans: the trouble with talking about disparities. *J Health Commun* 2014:1-8.

[38] Friedman AL, Uhrig J, Poehlman J*, et al.* Promoting sexual health equity in the United States: implications from exploratory research with African-American adults. *Health Educ Res* 2014;**29**(6):993-1004.

[39] Reynolds JP, Pilling M, Marteau TM. Communicating quantitative evidence of policy effectiveness and support for the policy: three experimental studies. *Soc Sci Med* 2018;**218**:1-12.

[40] Jacoby WG. Issue framing and public opinion on government spending. *Am J Pol Sci* 2000:750-767.

[41] Wallack L, Dorfman L. Media advocacy: a strategy for advancing policy and promoting health. *Health Educ Q* 1996;**23**(3):293-317.

[42] Roth AL, Dunsby J, Bero LA. Framing processes in public commentary on US federal tobacco control regulation. *Soc Stud Sci* 2003;**33**(1):7-44.

[43] Niederdeppe J, Gollust SE, Jarlenski MP*, et al.* News coverage of sugar-sweetened beverage taxes: pro- and antitax arguments in public discourse. *Am J Public Health* 2013;**103**(6):e92-98.

[44] National Cancer Institute Behavioral Research Program. NCI-CRUK Sandpit Workshops. 2017. Accessed December 10, 2020. Available from: https://cancercontrol.cancer.gov/brp/hbrb/sandpit.html

[45] Braun V, Clarke V. Thematic analysis. In: Cooper H, Camic PM, Long DL*, et al.*, eds. *APA Handbook of Research Methods in Psychology: Research Designs: Quantitative, Qualitative, Neuropsychological, and Biological*. Washington, DC: American Psychological Association 2012.

[46] Lee JG, Conway DI, Carey RN*, et al.* Dataverse file: Candidate frames tested in pilot project. 2020. Accessed December 10, 2020. Available from: https://dataverse.unc.edu/file.xhtml?fileId=7513206

[47] Faul F, Erdfelder E, Lang AG*, et al.* G*Power 3: a flexible statistical power analysis program for the social, behavioral, and biomedical sciences. *Behav Res Methods* 2007;**39**(2):175-191.

[48] Jeong M, Zhang D, Morgan JC*, et al.* Similarities and differences in tobacco control research findings from convenience and probability samples. *Ann Behav Med* 2019;**53**(5):476-485.

[49] Brandon DM, Long JH, Loraas TM*, et al.* Online instrument delivery and participant recruitment services: emerging opportunities for behavioral accounting research. *Behavioral Research in Accounting* 2013;**26**(1):1-23.

[50] Brehm SS, Brehm JW. Psychological Reactance: A Theory of Freedom and Control. Academic Press 1981.

[51] Hall MG, Sheeran P, Noar SM*, et al.* Reactance to Health Warnings Scale: Development and Validation. *Ann Behav Med* 2016;**50**(5):736-750.

For peer review only - http://bmjopen.bmj.com/site/about/guidelines.xhtml

[52] Hayes AF. My macros and code for SPSS and SAS: OMEGA. 2019. Accessed December 10, 2020. Available from: http://afhayes.com/spss-sas-and-r-macros-and-code.html

[53] Cohen J, Cohen P, West SG, *et al.* Applied Multiple Regression/Correlation Analysis for the Behavioral Sciences. 3rd ed. Mahwah, NJ: Lawrence Erlbaum 2013.

[54] Smith CE, Hill SE, Amos A. Impact of population tobacco control interventions on socioeconomic inequalities in smoking: a systematic review and appraisal of future research directions. *Tob Control* 2020.

[55] Oliver K, Cairney P. The dos and don'ts of influencing policy: a systematic review of advice to academics. *Palgrave Commun* 2019;**5**(1):21.

[56] Wagenaar AC, Tobler AL, Komro KA. Effects of alcohol tax and price policies on morbidity and mortality: a systematic review. *Am J Public Health* 2010;**100**(11):2270-2278.

[57] Bennett R, Zorbas C, Huse O, *et al.* Prevalence of healthy and unhealthy food and beverage price promotions and their potential influence on shopper purchasing behaviour: A systematic review of the literature. *Obes Rev* 2020;**21**(1):e12948.

[58] Pimpin L, Sassi F, Corbould E, *et al.* Fiscal and Pricing Policies to Improve Public Health: A Review of the Evidence. London, UK: Public Health England 2018. Accessed December 10, 2020. Available from: https://assets.publishing.service.gov.uk/government/uploads/system/uploads/attachment_data/file/743118/Fiscal_and_Pricing_Policies_report_FINAL.pdf

[59] Martin L, Bauld L, Angus K. Rapid evidence review: The impact of promotions on high fat, sugar and salt (HFSS) food and drink on consumer purchasing and consumption behaviour and the effectiveness of retail environment interventions. Edinburgh: NHS Health Scotland 2017.

[60] Whitehead R, Brown L, Riches E, *et al.* Rapid evidence review: Strengths and limitations of tobacco taxation and pricing strategies. Edinburgh: NHS Health Scotland 2018.

[61] World Health Organization Regional Office for Europe. Using price policies to promote healthier diets. Copenhagen: WHO Regional Office for Europe 2015.

[62] Dorfman L. Talking about sugar sweetened-beverage taxes: will actions speak louder than words? *Am J Prev Med* 2013;**44**(2):194-195.

[63] Dorfman L, Krasnow ID. Public health and media advocacy. *Annu Rev Public Health* 2014;**35**:293-306.

[64] Diepeveen S, Ling T, Suhrcke M, *et al.* Public acceptability of government intervention to change health-related behaviours: a systematic review and narrative synthesis. *BMC Public Health* 2013;**13**:756.

[65] Antin TMJ, Annechino R, Hunt G, *et al.* The gendered experience of smoking stigma: implications for tobacco control. *Crit Public Health* 2017;**27**(4):443-454.

For peer review only

STROBE 2007 (v4) Statement—Checklist of items that should be included in reports of *cross-sectional studies*

As there is no applicable checklist for a factorial design, we are utilizing the STROBE statement.

Section/Topic	Item #	Recommendation	Reported on page #
**Title and abstract**	1	(*a*) Indicate the study's design with a commonly used term in the title or the abstract	1
		(*b*) Provide in the abstract an informative and balanced summary of what was done and what was found	2
**Introduction**			
Background/rationale	2	Explain the scientific background and rationale for the investigation being reported	4-5
Objectives	3	State specific objectives, including any prespecified hypotheses	5
**Methods**			
Study design	4	Present key elements of study design early in the paper	5
Setting	5	Describe the setting, locations, and relevant dates, including periods of recruitment, exposure, follow-up, and data collection	5-8
Participants	6	(*a*) Give the eligibility criteria, and the sources and methods of selection of participants	7-8
Variables	7	Clearly define all outcomes, exposures, predictors, potential confounders, and effect modifiers. Give diagnostic criteria, if applicable	8-9
Data sources/ measurement	8*	For each variable of interest, give sources of data and details of methods of assessment (measurement). Describe comparability of assessment methods if there is more than one group	8-9 (+ codebook in repository)
Bias	9	Describe any efforts to address potential sources of bias	9
Study size	10	Explain how the study size was arrived at	7
Quantitative variables	11	Explain how quantitative variables were handled in the analyses. If applicable, describe which groupings were chosen and why	9-10
Statistical methods	12	(*a*) Describe all statistical methods, including those used to control for confounding	9-10
		(*b*) Describe any methods used to examine subgroups and interactions	9-10
		(*c*) Explain how missing data were addressed	N/A – minimal sporadic missingness

For peer review only

		(*d*) If applicable, describe analytical methods taking account of sampling strategy	N/A
		(*e*) Describe any sensitivity analyses	N/A
**Results**			
Participants	13*	(a) Report numbers of individuals at each stage of study—eg numbers potentially eligible, examined for eligibility, confirmed eligible, included in the study, completing follow-up, and analysed	N/A
		(b) Give reasons for non-participation at each stage	N/A
		(c) Consider use of a flow diagram	N/A
Descriptive data	14*	(a) Give characteristics of study participants (eg demographic, clinical, social) and information on exposures and potential confounders	14
		(b) Indicate number of participants with missing data for each variable of interest	N/A – Minimal sporadic missingness
Outcome data	15*	Report numbers of outcome events or summary measures	15-18
Main results	16	(*a*) Give unadjusted estimates and, if applicable, confounder-adjusted estimates and their precision (eg, 95% confidence interval). Make clear which confounders were adjusted for and why they were included	15-18
		(*b*) Report category boundaries when continuous variables were categorized	Codebook
		(*c*) If relevant, consider translating estimates of relative risk into absolute risk for a meaningful time period	N/A
Other analyses	17	Report other analyses done—eg analyses of subgroups and interactions, and sensitivity analyses	N/A
**Discussion**			
Key results	18	Summarise key results with reference to study objectives	19
Limitations	19	Discuss limitations of the study, taking into account sources of potential bias or imprecision. Discuss both direction and magnitude of any potential bias	19-20
Interpretation	20	Give a cautious overall interpretation of results considering objectives, limitations, multiplicity of analyses, results from similar studies, and other relevant evidence	20-21
Generalisability	21	Discuss the generalisability (external validity) of the study results	19-20
**Other information**			
Funding	22	Give the source of funding and the role of the funders for the present study and, if applicable, for the original study on which the present article is based	3

*Give information separately for cases and controls in case-control studies and, if applicable, for exposed and unexposed groups in cohort and cross-sectional studies.

For peer review only

Note: An Explanation and Elaboration article discusses each checklist item and gives methodological background and published examples of transparent reporting. The STROBE checklist is best used in conjunction with this article (freely available on the Web sites of PLoS Medicine at http://www.plosmedicine.org/, Annals of Internal Medicine at http://www.annals.org/, and Epidemiology at http://www.epidem.com/). Information on the STROBE Initiative is available at www.strobe-statement.org.

For peer review only
